# Beyond classical metrics: Generalizability theory across psychophysiological modalities

**DOI:** 10.1016/j.ijpsycho.2026.113321

**Published:** 2026-01-09

**Authors:** Harold A. Rocha, Amanda Holbrook, Greg Hajcak, Andreas Keil, Philippe Rast, Julian F. Thayer, Edelyn Verona, Walter P. Vispoel, Peter E. Clayson

**Affiliations:** a Department of Psychology, University of South Florida, Tampa, FL, USA; b School of Education and Counseling, Santa Clara University, Santa Clara, CA, USA; c Department of Psychology, University of Florida, Gainesville, FL, USA; d Department of Psychology, University of California-Davis, Davis, CA, USA; e Department of Psychology, Ohio State University, Columbus, OH, USA and Department of Psychological Science, University of California, Irvine, Irvine, CA, USA; f Department of Psychological and Quantitative Foundations, University of Iowa, Iowa City, IA, USA

**Keywords:** Generalizability theory, Psychophysiological measurement, Individual differences, Psychometric reliability, Multilevel models, Difference scores

## Abstract

Psychophysiological research relies on biological measures to understand cognitive, affective, and behavioral processes, but the utility of these measures for studying individual differences depends on their psychometric reliability. Traditional reliability methods, such as classical test theory, often fail to account for the multiple sources of variance inherent in psychophysiological data. Generalizability theory (GT) provides a robust, multifaceted approach to reliability estimation by decomposing variance across multiple facets, such as trials, tasks, and sessions. This article introduces GT to psychophysiological researchers, detailing its advantages over classical approaches and demonstrating its application to a variety of psychophysiological modalities: event-related potentials (ERPs), electroencephalography (EEG), electrodermal activity (EDA), electromyography (EMG), and electrocardiography (ECG). We outline the two-phase process of GT: generalizability (G) studies, which quantify variance components, and decision (D) studies, which optimize reliability within study designs intended for specific purposes. Psychometric formulas are provided for estimating indices of generalizability, dependability, and measurement error for numerous designs, including designs based on difference scores. Additionally, we discuss best practices for variance component estimation, highlighting the advantages of multilevel modeling in handling unbalanced data and non-normal distributions, typical of psychophysiological data. By applying GT, researchers can enhance the replicability and interpretability of psychophysiological measures, ultimately strengthening their ability to link biological signals to psychological constructs. This framework represents a necessary evolution in psychophysiological science, ensuring that biological measurements are grounded in fundamental psychometric principles.

## 1. Introduction

Psychophysiological research seeks to understand psychological phenomena as manifested across diverse biological measures. Biological measures can offer insights into the connections among cognitive, motor, affective, and sensory systems. A goal of some psychophysiological research is to understand how individual differences in biological measurements relate to individual differences in psychological traits or states, but the suitability of any measurement for studying individual differences depends on its psychometric reliability (Clayson, 2024b). If a measure is not reliable, it cannot be valid. The use of measurements, biological or otherwise, with poor psychometric characteristics increases the likelihood of missing true phenomena and finding non-replicable effects (Clayson et al., 2021c; Heindorf et al., 2025; Loken and Gelman, 2017). Any statistical prediction is limited by the reliability of the measurements involved (Thorndike, 1951). Therefore, identifying biological measurements with adequate psychometric characteristics will improve the potential utility of these measures in studies of individual differences.

Reliability assessments of psychophysiological data often use classical test theory, which assumes that an observed score represents a combination of a true score and undifferentiated measurement error and that true and error scores are independent of each other (Brennan, 2010; Cronbach et al., 1972; Cronbach et al., 1963; Le et al., 2009; Lord and Novick, 1968; Spearman, 1910; Vispoel et al., 2018). While classical test theory typically considers a single index of measurement error, generalizability theory^1^ (GT) facilitates the estimation of multiple sources of variability, including multiple sources of error variance, and requires specifying the universe of generalization that bounds the intended inferences. An understanding of sources of variance is important to identify ways to optimize measurements for studying individual differences (Clayson and Miller, 2017b). Psychometric work has long emphasized that optimizing a measure for individual differences begins with systematic decomposition of its score variance, and only after observed-score variance is dissected into identifiable factors can the unpredictable change portion of measurement variance be considered true error (Thorndike, 1951). Because psychophysiological measurements are typically collected across many trials, sensors, and tasks, variability from such sources can easily obscure genuine between-subject differences. Reducing extraneous variability is therefore essential for investigating individual differences. GT enables a comprehensive examination of sources of variance contributing to the variability of observed scores, yielding a rich understanding of measurement characteristics. GT is especially valuable when there are multiple identifiable sources of measurement variance that cannot be easily accounted for using classical test theory. This is typical in studies that use psychophysiological measurements, which often involve many identifiable sources of variance (e.g., trials, occasions, tasks/experimental paradigms, events, hardware, participant characteristics).

The present article provides an accessible tutorial and practical guide to applying GT to psychophysiological measurements, with the goal of helping researchers leverage GT’s flexible, multifaceted framework when evaluating and optimizing psychometric reliability. We first introduce GT and then provide an overview of how to apply GT to typical data structures encountered in psychophysiology with an eye toward optimizing measurements for psychometric reliability. Although published developments of GT for studies of psychophysiological measurements have emphasized event-related brain potentials (ERPs; Baldwin et al., 2015; Carbine et al., 2021; Clayson et al., 2021a; Clayson et al., 2021c; Clayson et al., 2021d; Clayson and Miller, 2017a; Di Nocera et al., 2001; Hill et al., 2023), the present tutorial extends applications of GT to additional psychophysiological modalities, including oscillatory phenomena in electroencephalographic (EEG) recordings, electrodermal activity (EDA), electrocardiographic data (ECG), and electromyographic (EMG) measures. Nonetheless, the principles described herein apply to a broad range of psychophysiological modalities (e.g., functional magnetic resonance imaging [fMRI], electrogastrographic [EGG] recordings) and beyond (e.g., response times, accuracy).

## 2. Psychometric reliability

The term “reliability” has different meanings across fields of study and generally refers to a quantification of (in)consistencies of measurements. For instance, in physics or engineering, reliability often characterizes how consistently an instrument can measure a physical quantity (Brandmaier et al., 2018). However, in education or psychology, the concept of reliability typically concerns how well scores from a measure consistently distinguish differences among individuals or within the same individual over time (Cronbach, 1947). In both contexts, some notion of replication is essential, such as repeated calibrations of a device (physics, engineering) or repeated measurements across items or occasions (education, psychology). Still, the specific indices used to estimate reliability can differ. In psychology, it is common to use test-retest correlations, as well as single-occasion methods (e.g., coefficient alpha, split-half correlations), to estimate reliability of scores. Meanwhile, standard errors of measurement (SEMs) can offer an alternative lens that some authors argue (e.g., Cronbach, 1947), even within the field of psychophysiology (Luck et al., 2021), can be more informative for practical inferences than reliability coefficients.

This distinction is important because reliability in physics or engineering directly speaks to instrument functioning under stable conditions, whereas reliability in psychology usually addresses how consistently a measure captures inter- or intra-individual differences in one or more latent constructs (Cronbach, 1947). In education and psychology, psychometric reliability is primarily about whether the measurements are precise “enough” for discriminating among participants (i.e., the intended objects of measurement). In the present article, we use this latter definition of reliability (i.e., psychometric or score reliability), reflecting the focus on consistency of person-level scores across items/trials, forms, occasions, etc. Nonetheless, to clarify the scope of reliability estimation procedures for diverse research applications, we acknowledge that different fields rely on their own reliability indices and that standard errors of measurement can sometimes provide more practical metrics for interpreting measurement precision.

Characterizing the reliability of observed scores provides context for what can be learned from psychophysiological measurements in basic and applied settings (Clayson, 2025). When the reliability of scores is low, observed between-person differences largely reflect random fluctuations and context-specific noise rather than stable between-person variance, which distorts associations with external correlates and can lead to misleading findings (Thorndike, 1951). Additionally, such measurement error in scores with low reliability reduces statistical power (Ellis, 2013; Nicewander and Price, 1983; Williams et al., 1995) and contributes to unstable, non-replicable findings by inflating uncertainty and obscuring true effects (Clayson et al., 2021c; Heindorf et al., 2025; Loken and Gelman, 2017). These issues are relevant for studies that use psychophysiology methods because many laboratory paradigms were designed to yield robust within-person experimental effects (Clayson, 2025); as a result, tasks can produce robust experimental effects while yielding low between-person variance and ultimately poor reliability, a pattern termed the “reliability paradox” (Hedge et al., 2018). For studies of individual differences, the reliability paradox is problematic because it means that a task can appear effective for detecting experimental effects while producing unreliable scores, making it poorly suited for correlational, predictive, or biomarker research. Accordingly, routine evaluation and transparent reporting of reliability are prerequisites for any study that aims to explain, predict, or intervene on a measure of individual differences, whether the goal is basic theory building or applied translation (Clayson, 2024b, 2025).

## 3. Overview of generalizability theory

GT’s approach to estimating reliability comprises two phases. During the first phase, called a generalizability (G) study, data are analyzed such that the variance of the “signal” of interest is quantified relative to the additional contributions of competing sources of measurement variance (“noise;” effects of trials/items,^2^ occasions, tasks, etc.). Variance components are estimated to quantify the specific contribution of particular sources of overall score variability. This estimation of variance components can be challenging, especially when features of the data are not normally distributed or when data are unbalanced. Approaches for estimating variance components of psychophysiological data are discussed in detail in section 7. The second phase, called a decision (D) study, uses the estimated variances to understand the impact of each source on the observed scores so researchers can plan, for example, how many trials, sessions, or sensors are likely needed to achieve a target level of reliability. The D study can optimize measurements by calibrating multiple sources of measurement error to understand their effects on psychometric reliability. While each step is traditionally referred to as a “study,” they are merely separate phases of analyses.

### 3.1. Example study design

An investigation by Riesel et al. (2013) provides a ready example that illustrates the relevant considerations for psychometric analysis and a starting point for discussing GT (for a direct replication, see Clayson et al., 2025b). The purpose of the study was to examine the psychometric characteristics of error-related negativity (ERN) and error positivity (Pe) across three commonly used tasks (Stroop, flanker, go/no-go). The ERN is generally interpreted as an index of early error detection (for review, see Larson et al., 2014), and Pe is generally interpreted as an index of conscious error awareness (for review, see Overbeek et al., 2005). This example study represents a departure from a typical ERN/Pe study which uses a single task (e.g., just Stroop) in that it included the same ERP components recorded from three paradigms. The use of this study illustrates GT’s flexible framework and how it can be leveraged to understand the contribution of multiple sources of variance in observed scores.

### 3.2. Generalizability (G) study

A beginning step in a GT analysis is to identify the *universe score* (italicized words are defined in Table 1) to be estimated, as this provides the scaffolding for building the necessary reliability algorithms. The universe score is the signal of interest, analogous to classical test theory’s true score, but GT’s semantic distinction respects that any observed score belongs to many possible universes and that reliability depends on the universe (i.e., context[s] or operationalization[s]) of interest (Cronbach et al., 1972). The researcher then defines the *objects of measurement* and the population from which they are drawn. For an example study involving ERN or Pe scores from a flanker task, the objects of measurement might be healthy adults, college undergraduates, women with depressive disorders, children of people with schizophrenia, etc. Next, the *universe of admissible observations* is identified that would encompass all possible *conditions* for the measurement facets of interest (trials, occasions, etc.).

Measurement facets of interest represent potential sources of measurement error analogous to factors in an analysis of variance (ANOVA). These facets vary in terms of how many conditions of measurement are sampled. Variance estimates of these facets are calculated based on their structure within a given experiment. In the example ERN/Pe study (see section 3.1), facets could include event type (with conditions of measurement specified as correct and error response types), trials, administration occasions, and tasks (with conditions of measurement possibly specified as Stroop, flanker, and go/no-go tasks).^3^ Facets are *fixed* or *random* depending on whether researchers aim to limit their inferences to specific, predefined conditions (i.e., fixed) or seek to generalize their findings beyond those specific conditions to a larger population or broader contexts (i.e., random). In the study being described, an example of a fixed facet is event type given that error-monitoring tasks only include responses classified as either correct (correct-related negativity [CRN]) or incorrect (ERN). In contrast, the *trials* (i.e., trial 1, trial 2, trial 3, etc.) and *occasions* (e.g., two sessions one week apart) facets would be considered random as there is typically no special meaning attributed to any particular trial response or administration session; that is, the ERN component score for the first error is entirely exchangeable with the score for the fifth or fiftieth error.^4^ The first error is not meaningfully aligned across participants, and similarly those responses are considered exchangeable (interchangeable draws from the same underlying distribution) across occasions. The task facet (e.g., flanker vs. go/no-go) could be either fixed or random. If a researcher is only interested in generalizing to the three tasks sampled, then the facet should be fixed. However, if the three tasks are considered only a sample of all possible tasks used to record ERN or Pe (e.g., Park et al., 2024), then the task facet could be treated as random.^5^ Together, the facets of interest are determined when identifying the universe of admissible observations, which represents the scope of conditions considered across relevant designs as a whole.

After identifying the facets of interest and whether they should be fixed or random, a *generalizability (G) study* is performed to isolate and estimate variance components (for a description of variance estimation procedures, see section 7). The sources of variance modeled would include all relevant measurement facets within the universe of admissible observations and their associated interactions. In the example study (see section 3.1), which uses a comprehensive mixed design that includes both random and fixed facets, variance components associated with persons, trials, and occasions are considered random, and variance components associated with event types and tasks are considered fixed. The highest order and most complex interaction (*Persons* × *Trials:Event Types* × *Tasks* × *Occasions*)^6^ in this example would be confounded with any residual measurement error unaccounted for by other terms within the design (Shavelson and Webb, 1991). An examination of the variance components for the facets will characterize the relative size of their contributions to observed single-trial ERP component scores.

### 3.3. Decision (D) study

The estimated variance components from the G study are then applied in a *decision (D) study* analysis to estimate reliability for a particular purpose within a particular context. A D study analysis requires defining the *universe of generalization*, which, unlike the universe of admissible observations (all potential facet conditions), specifies the conditions of the facets that the researcher wants to generalize to, which do not necessarily include all original facets. GT formalizes that reliability is always defined relative to a specified universe of generalization, so the facets included explicitly determine the boundary conditions under which scores can be interpreted. In the example study, ERP scores are derived from a limited number of trials within the universe of admissible observations. However, if trials are treated as a random facet, variance components estimated from the sampled scores can be used to represent the extent to which results can be generalized to any given number of trials. Considering that tasks and event types were treated as fixed facets in this example, the intent is to generalize only to the observed conditions for the two random facets (trials and occasions). Therefore, a purpose of the D study analysis could be to determine the number of trials needed to achieve a certain threshold of reliability within a single occasion. Conceptually, a D study takes what is known about the variances of measurement errors across many circumstances and uses them to estimate reliability for a specific subset of contingencies (e.g., a specific number of trials).

The appropriate reliability coefficient to estimate in a D study analysis depends on the type of inference a researcher wants to make. A question that a researcher might ask is the extent to which ERP scores generalize across the entire universe of generalization—that is, how consistent they are across all trials and occasions within the targeted domains when taking into account both within-person effects and differences among facet condition means. To answer this question, GT offers a *dependability coefficient (ϕ)*, which includes these sources of variance when estimating the consistency of scores. In the example study, a dependability coefficient might be estimated to quantify the overall consistency of ERPs across all possible trials and occasions within the designated universe.

Alternatively, a *generalizability coefficient $\left({E\rho }^{2}\right)$* is less stringent and reflects the extent to which within-person score variance contributes to total observed score variance controlling for means among facet condition scores. As such, in the example study, the generalizability coefficient would reflect the extent to which scores for trials and occasions generalize to the broader domains from which they were drawn independent of the absolute levels of scores. Therefore, between-trial and between-occasion mean differences in variance do not count against the generalizability coefficient (except for their interactions with persons), but they *do* impact the dependability coefficient.

#### 3.3.1. Defining the error

**Fig. 1.**
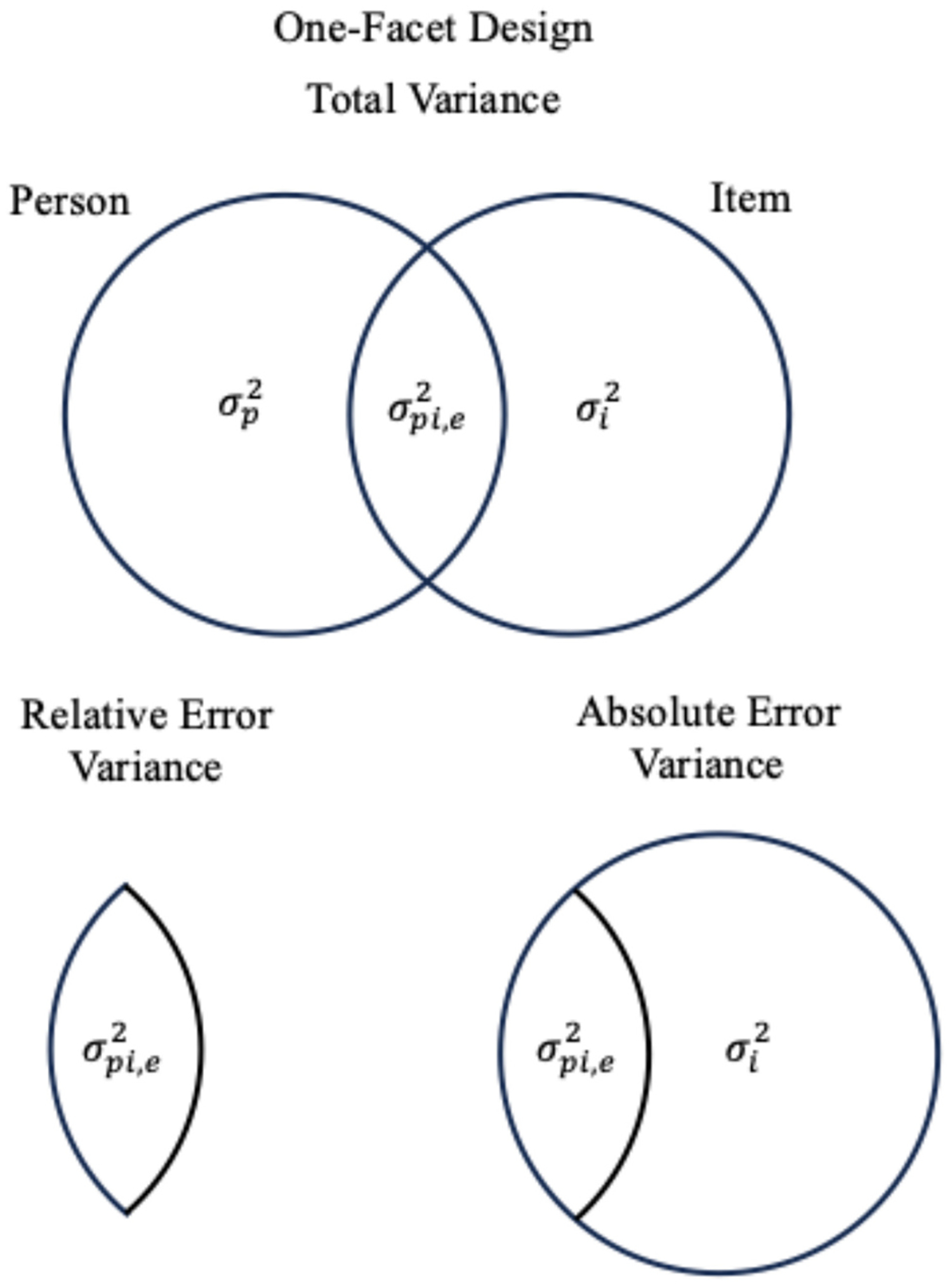
Venn diagrams show how total observed score variance is allocated in a one-facet (person × item) design. Items are treated as trials in the body of the article. Each circle represents a facet, and overlapping areas show interactions among facets. At the top of each diagram is the total observed score variance, while each circle depicts that facet’s contribution to the overall variance. The figure presented is intended strictly for convenience and conceptual purposes. The magnitude of error components suggested by the areas in the diagrams does not reflect proportions of explained total score variance typically found in practice in which person-related effects generally exceed those for facet condition means. These diagrams also highlight the key distinction between generalizability coefficients $\left(\text{E}\rho^{2}\right)$, which focus on relative error variance, and dependability coefficients (ϕ), which use absolute error variance. Generalizability coefficients only consider error sources that affect participants’ relative rankings, so for a one-facet design, only the variance overlapping with the “Person” circle—in this case, the person–item interaction $\left(\sigma_{pi,e}^{2}\right)$—is incorporated into the reliability estimate (see Table 1 for details). In contrast, dependability coefficients include all sources of variance that influence observed scores (absolute error). As a result, they account for both the item variance $\left(\sigma_{i}^{2}\right)$ and the person–item interaction variance $\left(\sigma_{pi,e}^{2}\right)$ in a one-facet design.

**Fig. 2.**
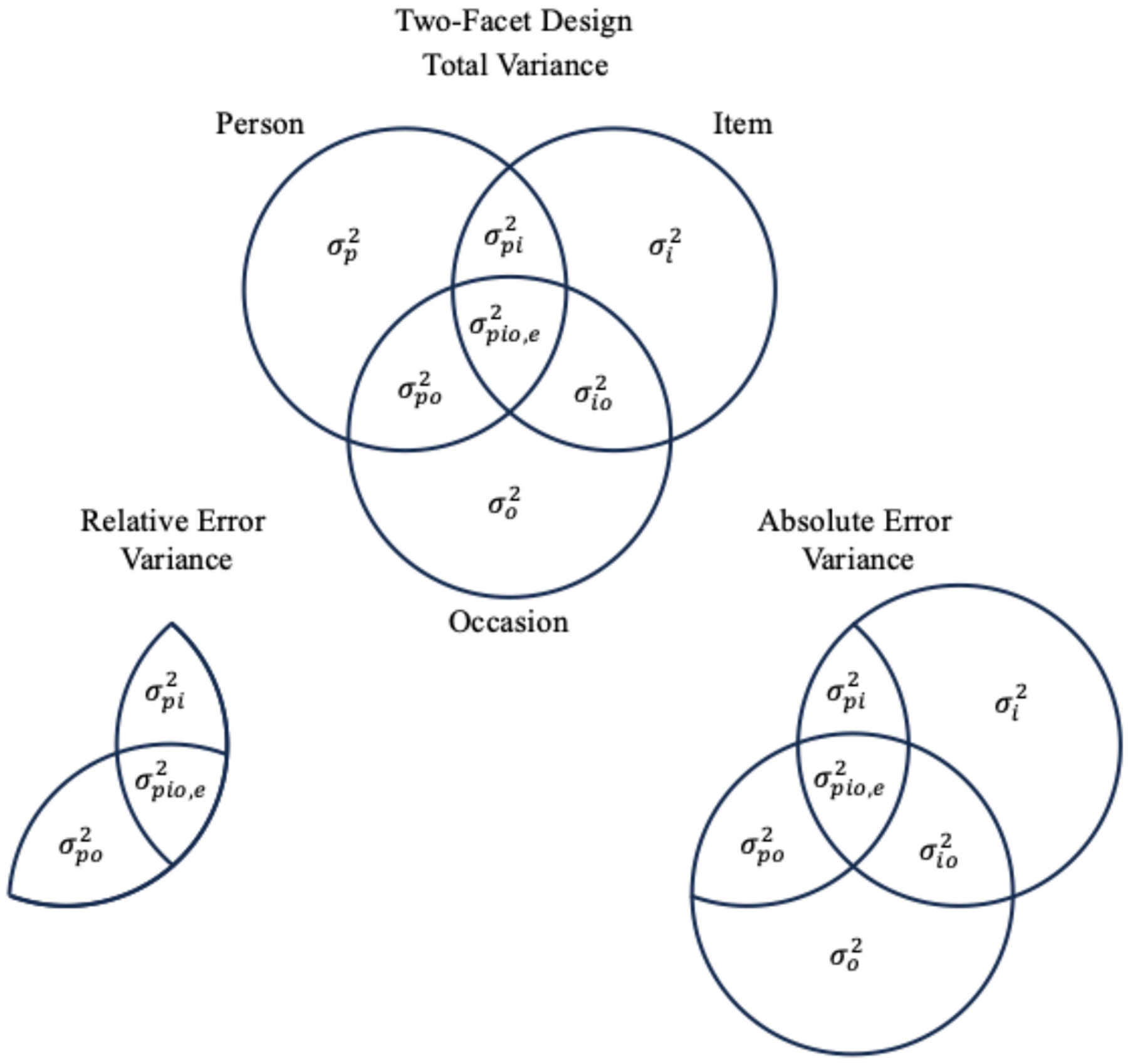
Venn diagrams show a two-facet design (person × item × occasion). Items are treated as trials in the body of the article. At the top is the total observed score variance, and each circle represents a specific facet; areas where circles overlap show the interactions among these facets. The figure presented is intended strictly for convenience and conceptual purposes. The magnitude of error components suggested by the areas in the diagrams does not reflect proportions of explained total score variance typically found in practice in which person-related effects generally exceed those for facet condition means. Generalizability coefficients $\left(\text{E}\rho^{2}\right)$ focus on how error variance influences participants’ relative standings (i.e., relative error). Consequently, only the variance overlapping with the “Person” circle contributes to reliability in this framework. For the coefficient of trial equivalence and stability (CES; see Table 1), the relevant sources of error variance include the person–item $\left(\sigma_{pi}^{2}\right)$ and person–occasion $\left(\sigma_{po}^{2}\right)$ interactions, as well as the three-way interaction $\left(\sigma_{pio,e}^{2}\right)$. By contrast, the dependability (ϕ) CES incorporates all facets and their interactions, reflecting absolute error, in its estimate of reliability. Item variance $\left(\sigma_{i}^{2}\right)$, occasion variance $\left(\sigma_{o}^{2}\right)$, and their interaction $\left(\sigma_{io}^{2}\right)$ are typically smaller than those for relative error, but the figure shows equal contributions for the sake of readability.

For both dependability and generalizability coefficients, a ratio is calculated between the universe score variance and the universe score variance in addition to all relevant error variances (e.g., attributable to measurement facets; example formulas are provided in Tables 2 and 3). See Fig. 1 for a one-facet (*Persons* × *Items*) design or Fig. 2 for a two-facet (*Persons* × *Items* × *Occasions*) design. While the dependability coefficient is thought of as an absolute measure of consistency or measurement accuracy, the generalizability coefficient is a relative measure of consistency, because it is concerned with the expected variance of the universe score when controlling for the effects of the facets of interest unrelated to person-specific variance (i.e., only variances due to persons and the interactions between persons and measurement facets).

The error variance term in calculating each coefficient differs (see Figs. 1 and 2). Generalizability coefficients are computed using *relative error variance*, which only includes person-specific variance components. The GT formulas for one- and two-facet designs are shown in Tables 2 and 3. In a two-facet *Persons* × *Trials* × *Occasions* study, the generalizability coefficient would include as error variance the idiosyncratic differences between individuals across trials $\left(\sigma_{pi}^{2}\right)$, across occasions $\left(\sigma_{po}^{2}\right)$, and across combinations of trials and occasions $\left(\sigma_{pio,e}^{2}\right)$. It would not include systematic trial or occasion differences outside of their interactions with persons, as these facet condition means would be constants—therefore, not impacting the ranking of individuals. Trial and occasion mean differences do not contribute to the generalizability coefficient because the variance contribution is functionally the same for all persons. The result is that a generalizability coefficient expresses reliability that, on average, could be expected within any combination of conditions that are specified within a given design (in this case, “for a given number of trials and occasions”).

Relative error variance is contrasted with *absolute error variance*, as illustrated in Figs. 1 and 2. *Absolute error variance* includes mean-score level variances and is used to generate the dependability coefficient. Absolute error variance, in the simplified ongoing ERP example shown in Fig. 2, includes the unique variance attributable to trials $\left(\sigma_{i}^{2}\right)$, occasions $\left(\sigma_{o}^{2}\right)$, and their interaction $\left(\sigma_{io}^{2}\right)$. In essence, absolute error variance defines error as any variance in the design not unique to the universe score. It is, in effect, the amount of error variance to be expected when accounting for facet conditions as a whole. When score means do not differ between levels of all facets of interest, the absolute error variance is equivalent to relative error variance. For example, if ERP scores do not systematically differ between trials or occasions, then the absolute and relative error variances would not differ, and the dependability coefficient equals the corresponding generalizability coefficient.

#### 3.3.2. Types of reliability

In a design for objectively scored assessments that includes both items (trials here) and occasions (or sessions) as facets, the generalizability and dependability coefficients will include $\text{Persons}\times \text{Items}(\sigma_{pi}^{2}),\text{Persons}\times \text{Occasions}(\sigma_{po}^{2})$, and $\text{Persons}\times \text{Items}\times \text{Occasions}(\sigma_{pio}^{2})$ interaction terms. These terms can be used to answer questions common in psychometric research, including identifying the impact of specific-factor (e.g., internal reliability) and transient error (e.g., test-retest reliability) on observed score variance.

Specific-factor error is a systematic source of variance that is not from the construct of interest, such as cohort effects or, in our example, person-item interactions. Classical test theory’s single-occasion coefficient alpha and Spearman-Brown adjusted split-half coefficients are similar to GT’s generalizability *coefficient of equivalence* (CE) for a one-facet design for a one-session study (Cronbach, 1947), which quantifies the ratio of universe-score variance to universe score plus undifferentiated error variances. CEs can range from 0 to 1, with 1 indicating perfect equivalence. In classical test theory, a CE also could be used as an estimate of internal reliability when item-level scores are analyzed. CEs in a GT context differ from CEs in classical test theory involving parallel forms in that they reflect random rather than strict equivalence.

Transient error refers to variance that is unique to the individual and that varies across occasions possibly due to changes in the individual’s state unrelated to the construct of interest. GT can produce a *coefficient of stability* (CS) analogous to test-retest reliability by examining variance across sessions (Cronbach, 1947). CS can measure the extent to which ERP scores vary across administration occasions and range from 0 to 1, with 1 indicating perfect stability. The CS characterizes retest reliability of ERPs by typically treating “trials” as a fixed facet with the conditions for occasions corresponding to each study visit. A more thorough example of deriving CEs and CSs from a two-facet design using both trials and occasions can be seen elsewhere in assessments of ERP retest reliability (see Carbine et al., 2021; Clayson et al., 2021d).

In the ERP example study (see section 3.1), some variance in scores likely arises due to the measurement occasion, but if participants only attend one measurement occasion (i.e., participants only attended one study session in which they completed the experimental tasks), variance across occasions cannot be estimated (i.e., person [e.g., trait] and transient error [e.g., state] effects are confounded). Thus, occasion would serve as a *hidden facet* when reporting CEs. Similarly, trials or items (i.e., specific-factor effects) would be a hidden facet when reporting CSs, because person and trial/item effects are confounded. A facet is often hidden when only one condition for that facet has been sampled. In electrophysiological research, hidden facets often include hardware and experimental paradigms used to elicit a given ERP component, as relatively few studies compare component scores across conditions of these facets. If only one paradigm is administered, task-related variability cannot be estimated (i.e., is unidentifiable), and the resulting reliability is for the paradigm used. It is best to include as many relevant facets as possible when estimating variance components and reliability coefficients to avoid overestimating reliability.

Finally, a G coefficient that includes inter-person trial (or item) and occasion differences as measurement error rather than part of the universe score can be considered a combined coefficient of trial equivalence and stability over time for a *Persons* × *Trials* × *Occasions* design (see Table 3). The coefficient of trial equivalence and stability represents a ratio of universe-score variance over the sum of universe score, specific-factor error, transient error, and random-response error variances. Similar to CEs and CSs, coefficients of trial equivalence and stability range from 0 to 1, with 1 indicating perfect equivalence and stability. In an ERP context, coefficients of trial equivalence and stability can be interpreted as reliability estimates that better reflect the generalizability of scores across trials and occasions than would a CE or CS estimated from a one-facet, occasion only or trial only design (Le et al., 2009).

#### 3.3.3. Cut-score reliability

In the context of a D study, a *cut-score-specific dependability coefficient* can be used to determine the level of precision in identifying an individual’s standing relative to a specific threshold (e.g., clinical vs. healthy functioning). Among the ERP components investigated in the example study is ERN. People with generalized anxiety disorder (GAD) typically show larger ERN than healthy comparison participants (for review, see Hajcak et al., 2019). Determining values for cut-score-specific dependability coefficients at various ERN amplitudes may facilitate specifications of thresholds that could reliably differentiate people with GAD from healthy participants. Computationally, a cut-score-specific coefficient adds a cut score’s squared deviation from the estimated universe score mean to determine levels of agreement in observed score locations relative to the cut score over random replications of the measurement procedure (see Tables 2–5). When the cut score matches the scale mean, the cut-score dependability coefficient equals the global dependability coefficient, reflecting the average expected variance across all conditions for the observed score.

A common motivation for using cut scores is to distinguish clinical from healthy functioning. Establishing cut scores for this purpose requires normative data derived from a clearly defined reference distribution and a standardized measurement protocol^7^ (Clayson, 2024b, 2025; Clayson et al., 2021e). In educational, psychological, and neuropsychological assessment, normative interpretation is defensible only when administration and scoring are standardized to minimize extraneous variability; otherwise, an apparently extreme score can reflect procedural artifacts rather than true deviation in the construct of interest (Bigler and Dodrill, 1997; Russell et al., 2005). The normative sample must also be explicitly defined as the population to which interpretations are intended to generalize (Bayless et al., 1974; Mitrushina et al., 2005), and standardization samples are ideally collected via systematic stratification on key demographic variables (e.g., age, gender, education/occupation, geographic region) to support valid normative comparisons (Cicchetti, 1994). In contrast, samples of convenience can yield biased norms, add substantial error that is difficult to quantify, and produce inconsistent results, which severely limits the generalizability of cutoffs and increases the risk of misclassification when applied to new populations or settings (Andrade, 2021; Bayless et al., 1974). In psychophysiology research, these issues further motivate harmonized protocols (task parameters, acquisition settings, preprocessing, and scoring rules) so that normative databases and downstream cut-score decisions are anchored to a reproducible, well-specified measurement procedure (Clayson, 2024b, 2025). Critically, normative data intended for broad use should be derived from representative samples collected using protocols that are transparent, readily available, and standardized. Otherwise, cut scores risk being biased and misleading, which undermines generalizability and increases misclassification risk when thresholds are applied in new settings.

## 4. Psychophysiological data types

Traditional methods of estimating reliability often rely on assumptions that are violated by physiological data (Clayson, 2024b). For example, traditional computation of coefficient alpha requires an equal number of test items per person for estimating variance to calculate internal reliability, whereas physiological data typically produce a variable number of usable trials or data splits per person after artifact rejection procedures. Additionally, hypothesis tests for some reliability coefficients might assume that score values are normally distributed, despite some physiological measures being distributed differently.

An additional assumption that fails in some psychophysiological contexts is that the average of single-trial scores should equal the score computed from an averaged waveform; in practice, these two quantities can diverge when latency varies across trials. For example, the average of single-trial ERP peak amplitude scores is almost always higher than the peak amplitude score of the average of all ERP epochs, due to the variability in latency of the single-trial peaks. GT analyses, designed for parallel item task splits (i.e., exchangeable segments), can estimate the reliability of average peak amplitude scores (see Tables 4 and 5; see also Vispoel et al., 2022b). Using task splits instead of single-trial scores mitigates background EEG noise by averaging multiple trials within each split. In GT analyses, split-based scores can also serve as the unit of analysis. For example, ERP peak amplitudes averaged across 100-trial splits from a 500-trial dataset can be used to estimate reliability and determine optimal task length as a function of the number of splits. The appropriate split size depends on the data type and research objectives, and the size should be consistent across persons.

GT holds fewer assumptions than traditional methods, allowing differing data types to be adapted for reliability analyses with a few considerations: universes are unambiguously defined, facet conditions are independent, and scores are measured on equal-interval metrics (Cronbach et al., 1963). For non-normally distributed data, the distribution of the underlying scores can be specified when estimating variances, and multilevel models are well suited for doing so. The question of splits becomes relevant when splits are not independent of one another, such as when using overlapping time windows for estimating scores. In this scenario, score extraction for comparing changes in continuous data using moving time windows must avoid overlapping windows.

The appropriate reliability estimation procedure sometimes depends on the underlying distributions of the data, particularly when doing hypothesis testing or building confidence intervals. For example, hypothesis tests for some reliability methods might assume normally distributed scores, which may be appropriate for time-window mean amplitudes of ERP components but are less suitable for other psychophysiological measures, such as spectral power in EEG and frequency-domain heart rate variability (HRV). These measures often follow positively skewed or chi-square distributions due to their inherent properties, such as power being a squared amplitude value that cannot be negative. Ignoring these distributional differences in some contexts may lead to misestimation of variance components and misuses of reliability coefficients. Therefore, to ensure accurate psychometric evaluation within these contexts, reliability analyses must align with the statistical properties of the data, using models that accommodate their specific distributions. The following sections examine how this principle applies to spectral measures and nonlinear data, outlining appropriate reliability estimation methods for each.

### 4.1. EEG oscillations

There are various methods for processing and interpreting oscillatory signals in EEG. A common approach, the fast Fourier transform (FFT), decomposes neural time series amplitudes into constituent frequency bands (Hz) arrayed along the x-axis in a Fourier power spectrum, with amplitude power $\left({\mu \text{V}}^{2}\right)$ on the y-axis. This arrangement shows that low-frequency activity tends to contribute greater amplitude power to EEG signals than high-frequency activity. In fact, power spectra tend to reveal a negative exponential pattern/decay of power ranging from lowest to highest frequency.

The frequency bands in human EEG are associated with different states of consciousness and cortical phenomena. For instance, the alpha band, which ranges from 8 to 12 Hz in adults, is most active over sensory cortices deprived of novel sensory signals and is thought to measure sensory gating (Klimesch et al., 2007). The theta band, typically ranging from 4 to 7 Hz, is often associated with cognitive processes such as working memory, attention, and the encoding and retrieval of information (Klimesch, 1999; Valadez and Simons, 2018). It is prominently observed in frontal regions, reflecting the brain’s engagement in tasks requiring focused attention and the integration of cognitive functions. Similar to ERPs, activity in these spectral bands can be quantified by estimating either peak or mean amplitudes (in this case, amplitude power). The consideration of these scoring decisions is crucial for deriving reliable psychological measurements.

The amplitude power of a given frequency band varies by person (Berkhout and Walter, 1968; Rocca et al., 2014) and context (Klimesch, 1999), similar to the peak amplitude of ERP components. Additionally, the peak frequency of a given frequency band varies (Angelakis et al., 2004; Lindsley, 1939), much like ERP latencies do. An essential difference between them, however, is that, in many contexts, power is assumed to be amplitude squared and is thus always positive. This is because, conceptually, periodic oscillations include both regular negative and positive deflections, and amplitudes in either direction contribute to the magnitude of an oscillatory signal. This is not the case for time series data. For instance, gross muscle movement (e.g., neck movement) during EEG recording may result in large slow-wave positive or negative deflections that drastically impact ERP peak amplitudes. A P3 amplitude score might be 30 microvolts or – 100 microvolts, for example, depending on the movement artifacts it co-occurs with. When examining P3 amplitudes from many trials, if the noise is random, then amplitudes are normally distributed. However, an FFT of the same time series with gross-muscle artifacts would show increased power across the whole spectrum, with relative increases in the lower frequency bands, but values for a given point along the spectrum would never dip below zero.

Essentially, while ERP component amplitudes have virtually no floor or ceiling (due to the broad dynamic range of amplitudes that most EEG systems are equipped to record), EEG spectra expressed in microvolts squared have a noise floor, as they are bounded by zero. Even when power values are expressed in alternative units of measurement that encompass negative values (such as decibels), the noise floor is merely shifted while still producing an asymmetrical distribution bounded by some minimum value. Point estimates of a given frequency band, such as peak alpha power in EEG spectra, are therefore positively skewed and best represented using a chi-square distribution. In reliability analyses of frequency-domain scores, we recommend that variance components for scores from spectral data be estimated using multilevel models tailored to chi-square distributions.

#### 4.1.1. Frequency score extraction: periodic vs. aperiodic activity

Periodic activity in EEG waveforms, such as alpha spindles and steady-state evoked potentials, tends to form relative peaks along the frequency spectrum. In contrast, aperiodic “noise” (i.e., non-sinusoidal features of the waveform) is thought to drive the negative exponential curve (sometimes called the “noise floor”) of the spectrum. Oscillatory signals may be operationalized as either a total amplitude power (i.e., mean or peak amplitude power) or as a signal-band to noise-band ratio (SNR) of target frequency power to that of other frequency bands along the 1/f-seeming floor of the spectrum. SNRs of peak frequency power are often used in many experimental analyses because they exhibit good psychometric reliability (Rocha et al., 2020) and show more task-related variance than does total frequency-band power (Riels et al., 2020; Silva et al., 2021). Conversely, other researchers have examined individual differences in aperiodic activity itself as an index of cortical functioning by modeling the power distribution of the aperiodic noise floor itself (Donoghue et al., 2020b; Timpe et al., 2020). Recent studies parameterizing spectral data suggest that SNRs dependent on frequency-band ratios may sometimes conflate periodic and aperiodic contributions to frequency scores (Donoghue et al., 2020a; Ouyang et al., 2020), which presents a challenge to ontological interpretations of SNR findings.

Although researchers can now estimate aperiodic signals using the “fitting oscillations and one-over-f” (FOOOF) algorithm (Donoghue et al., 2020b), few studies have focused on the reliability of aperiodic activity itself (Pathania et al., 2021), and to our knowledge, the relative contribution of periodic and aperiodic variance to the reliability of scores of total frequency-band power has not been investigated. In future studies of oscillatory measures, researchers could apply generalizability theory by creating a “power score” facet with two levels: 1) periodic power (i.e., peak frequency power minus the estimated power of the noise floor) and 2) aperiodic power (the estimated power of the noise floor itself). This facet would help isolate variability relative to the 1/f-seeming curve and the relative peak above the curve. Additionally, interactions with other relevant facets (e.g., conditions, diagnostic groups) could be examined.

### 4.2. ECG

ECG records the electrical activity of the heart over time and is widely used to assess various cardiac parameters, including heart rate (HR), heart rate variability (HRV), and specific waveform features such as the P-wave, QRS complex, and T-wave, which correspond to successive stages of a given heart period (HP). These measures offer insights into autonomic nervous system functioning and its relation to psychological states. Just as with other psychophysiological measures, the reliability of ECG measurements is critical for their effective use in studying individual differences and understanding psychological phenomena. However, attempts to quantify reliability can be difficult due to the diversity of processing and quantification methods. Specifically, the many ways in which HRV can be estimated results in potential uncertainty regarding which distributions should be used when estimating variance components, which we have already discussed as a challenge to traditional reliability estimation methods. In this section, we discuss the implementation of GT to a study of HRV and consider different data types and distributions.

#### 4.2.1. Sample task for HRV: Trier Social Stress Task

To illustrate GT within the context of a social stress task, consider a study designed to examine HRV as a psychophysiological correlate of major depressive disorder (MDD). Participants engage in the Trier Social Stress Task (TSST; Kirschbaum et al., 1993), a commonly used task that involves a public speaking component followed by a mental arithmetic task in front of an audience, both of which are designed to elicit a stress response. HRV is recorded during baseline and task periods to examine how stress affects autonomic regulation. The aim of the study is to understand the reliability of HRV measurements in capturing individual differences in stress reactivity and recovery. Similar study designs in the ECG literature can be found in a systematic review conducted by Schiweck et al. (2019).

#### 4.2.2. ECG: generalizability (G) study

Similar to the EEG examples, a person’s observed HRV score is a composite of universe score (i.e., “true” participant) and measurement error effects. In this TSST example, the universe score represents a person’s HRV over all possible splits in the universe, considering the different phases of the TSST (baseline, public speaking, and mental arithmetic), with the population of interest being either healthy adults or adults with MDD. The facets of interest include the phase of the TSST (baseline, public speaking, and mental arithmetic) and splits (i.e., ECG segments used to estimate HRV), as well as the clinical group (healthy adults and adults with MDD).

The phase of the TSST facet includes three conditions: baseline, public speaking, and mental arithmetic. It can be treated as a fixed facet given that the TSST includes only these three phases of the task. The trials facet would be more aptly described as “splits”, reflecting the researcher-defined subdivision of a continuous recording into equal-length intervals (e.g., 1-min segments). Researchers determine the duration of each split, which should be long enough to capture meaningful activity (and average out noise) but still short enough to isolate discrete segments of measurement. The split facet is random because any split is considered representative of the overall response, and there is likely no inherent importance in the order of splits.^8^ The group facet includes two levels: “adults with MDD” and “adults with no known psychological disorder” (i.e., “healthy controls”; HC), which may be treated as a fixed facet because the study design specifically aims to compare these two groups^9^ (or stratified objects of measurement, Brennan, 2010, pp. 153–156).

HRV measures can be divided into three general categories: 1) global descriptive measures of the distribution of HPs, 2) measures of periodic features using spectral analyses, and 3) nonlinear measures of HRV based on dynamical systems modeling (Quigley et al., 2024). As mentioned in section 3.1, a challenge to applying reliability metrics to diverse data types is appropriately accounting for their underlying distributions. In the case of HRV, where the waveform itself is of secondary interest relative to its latency and periodicity, careful consideration must be given to data splits.

Data splits refer to dividing continuous time series data into segments or trials to evaluate their consistency. For example, HRV measures may be segmented into various intervals or windows, such as those used for calculating heart period (R-R intervals), root mean square of successive differences (RMSSD), or standard deviation measures (e.g., standard deviation of NN intervals). The longer a given split duration, the more variance it includes, due to the presence of very low frequency variation related to diurnal rhythms and increased occurrence of artifacts (e.g., due to coughing, speaking, or changes in posture). In the context of spectral measures of HRV, the assumption of stationarity is made. One form of this assumption is weak stationarity, in which the mean and variance of the underlying generator process does not change over time (Weber et al., 1992). Although there are several ways in which non-stationarity can be addressed, the appropriate choice of spectral method (i.e., autoregressive versus FFT) and use of appropriate methods for detrending the time series (e.g., higher-order polynomial or smoothness priors) can minimize violations of stationarity and the impact of aperiodic signals. Autoregressive spectral methods yield better spectral estimates with shorter data records (Kay and Marple, 1981), and appropriate detrending can minimize the effects of various trends in the data as well as reduce the influence of aperiodic signals (Tarvainen et al., 2002). As such, shorter split durations, which can reduce the effects of non-stationarity, and use of appropriate estimation algorithms and detrending, are generally recommended for most HRV estimates (Laborde et al., 2017). Each segment’s distribution can vary: Some measures, like RMSSD, might approximate normal distributions, while others, such as frequency domain measures, may exhibit positive skewness or chi-square distributions due to spectral power characteristics, as described in section 4.1.

The G study estimates variance components for each facet and their interactions. In this example, variance components include the main effects of person, split, the TSST phase, and their interactions. The same formulas shown in Tables 2 and 3 could be used, swapping trials for splits and occasions for phases. Data splits are of equal length for all participants (e.g., 1-min intervals) and as such use the same psychometric equations as those for trials. When the length of splits is uneven (e.g., 1 min, 2 min, 2.5 min), the split formulas shown in Tables 4 and 5 can be used.^10^ As always, the highest-order interaction, the three-way interaction in this case (*Persons* × *Splits* × *Phases*), is confounded with other unaccounted for error. As previously discussed, the estimation of variance will depend on the presumed distribution of scores. In terms of heart-rate variability, several measures exist, and the distributions of scores will depend on the quantification method used.

#### 4.2.3. ECG: decision (D) study

Using the variance components from the G study, a D study can assess the reliability of HRV measurements within the defined universe of generalization. In this example, the universe of generalization includes all HRV scores across different numbers of equal splits and TSST phases—in this context, analyses are conducted within group. For this example, the formulas specified in Table 2 for numbers of trials should be used, given that all participants within each group have equal-length splits.

The dependability coefficient assesses the consistency of HRV scores across these facets by including all of their variance components and those of their interactions, reflecting the precision of HRV measurements across these conditions and individuals. The generalizability coefficient focuses on the average consistency of HRV scores across the facet conditions. It controls for mean differences between splits and phases, providing a relative measure of consistency.

In this TSST example, the dependability coefficient would indicate how consistently HRV reflects stress reactivity and recovery across different splits and phases of the TSST for each group while encompassing differences in facet condition means. The generalizability coefficient would show the average consistency of HRV within specific conditions, such as during the baseline period for healthy adults or the public speaking phase for adults with MDD.

By applying GT to this example, researchers can optimize the study design, ensuring reliable HRV measurements that accurately capture individual differences in stress responses during the TSST. This detailed analysis highlights the utility of GT in enhancing the reliability and validity of psychophysiological measurements.

#### 4.2.4. Practical considerations

When applying GT to HRV data, the following steps can be helpful to consider. 1) Define splits clearly. Specify how data are segmented and averaged. For example, outline how HRV data are divided for measures like RMSSD or SDANN to ensure that the splits are representative of the broader measurement context. 2) Consider distributions. Recognize the distribution characteristics of different HRV measures. 3) Interpret reliability coefficients thoughtfully. GT reliability coefficients reflect the consistency of measurements across different conditions and time points. This insight is crucial for evaluating the robustness of HRV measures and ensuring that observed changes represent true physiological variations rather than random noise. By addressing these considerations, GT provides a comprehensive approach to accommodating the complexities inherent in HRV data and understanding its reliability.

### 4.3. EMG

Electromyographic (EMG) recordings reflect the electrical activity of muscles and are widely used to assess motor control, neuromuscular function, and physiological correlates of affective, cognitive, and sensory processes (Blumenthal et al., 2005). EMG signals can be recorded from various sites, such as the corrugator supercilii muscle or limb musculature. Despite its broad applicability, EMG poses unique measurement challenges arising from factors such as electrode placement, muscle fatigue, inter-individual differences in musculature, and environmental noise.

A common preprocessing step in EMG research involves rectifying the raw signal, transforming negative voltages to positive, thus facilitating the calculation of integrated or averaged EMG over specific time windows (Blumenthal et al., 2005). However, rectified EMG signals are positively skewed, with small, frequent amplitude fluctuations clustering near zero and occasional bursts during stronger muscle contractions producing heavy right tails (Basmajian and De Luca, 1985; Cacioppo et al., 1983). These distributional properties can violate assumptions underlying reliability analyses that assume normality.

#### 4.3.1. Sample task for EMG: neutral-predictable-unpredictable (NPU) threat task

The NPU threat task (Schmitz and Grillon, 2012) is used to examine threat across psychophysiological modalities (i.e., startle EMG, electrodermal activity). The NPU task involves a safe condition and conditions in which threat is predictable or unpredictable, with this task assessing reactivity to imminent threat (relevant to fear) and uncertain threat (relevant to anxiety and depression; Klumpp and Shankman, 2018). The NPU threat task is used in pharmacological and clinical studies and is an ideal task for translational studies (i.e., rodent studies of fear) that can incorporate a range of psychophysiological measures (Schmitz and Grillon, 2012). EMG startle potentiation is uniquely related to anxiety during predictable threat and to depression during unpredictable threat (Nelson and Hajcak, 2017).

#### 4.3.2. EMG: generalizability (G) study

GT can accommodate the multifaceted structure of EMG data by decomposing variance across multiple facets. For instance, researchers may consider facets such as muscle site (e.g., corrugator vs. *zygomaticus*), experimental condition (e.g., no threat, predictable threat, unpredictable threat), trial or data split, and occasion. A G study can identify the relative contributions of each facet, including facet interactions (*Persons* × *Muscle Sites, Persons* × *Conditions*, etc.), to observed score variance. Such information aids in determining whether differences in EMG scores primarily reflect participant-specific variance or facet-related measurement error. For example, a large *Persons* × *Muscle Sites* interaction might suggest that different muscle sites capture distinct aspects of EMG reactivity in different individuals.

It is important to note that estimated variance components should use the same data as those used for subsequent hypothesis testing. If hypothesis tests are run on raw scores, derive the variance components from raw scores; if the data are transformed (e.g., rectified EMG), estimate the variance components from the transformed values. Because rectified EMG amplitudes can differ markedly across participants, many laboratories standardize EMG by converting within-participant z scores to T scores. If hypothesis tests rely on these T-scored values, derive the variance components from the same T-scored data to maintain consistency between the G study and subsequent hypothesis tests.

#### 4.3.3. EMG: decision (D) study

A subsequent D study can then systematically adjust the levels of these facets (e.g., trials or splits, muscle sites) to achieve adequate reliability for individual differences research. As with other psychophysiological measures, multilevel modeling can be advantageous for estimating EMG variance components, given that it readily handles unbalanced data (e.g., differing numbers of valid EMG segments after artifact rejection) and can incorporate appropriate distributional assumptions.

Data from the NPU threat task could be used to model experimental condition as a fixed facet and occasion (two-week interval) as a random facet (a multivariate design could also be used and provide a univariate analysis for each experimental condition, see section 6). A CE from this two-facet design would provide a measure of internal reliability. Occasions would not be considered a hidden facet in this design because occasion-specific variance would be explicitly modeled in the overall design. Estimates of internal reliability (CEs) within the two-facet design (see Table 3) should provide more robust estimates of reliability than would those from one-facet designs because trials are averaged over occasions (see Clayson et al., 2021d). A CS from this two-facet design could provide an estimate of test-retest reliability over the specified two-week interval for any specified number of trials.

### 4.4. EDA

Electrodermal activity (EDA), encompassing both tonic skin conductance level (SCL) and phasic skin conductance responses (SCRs), provides an index of sympathetic nervous system activity (Boucsein, 2012; Society for Psychophysiological Research Ad Hoc Committee on Electrodermal Measures, 2012). EDA-based measures are frequently used in research on affective processes, attention, decision making, and various clinical phenomena.

EDA data are commonly decomposed into tonic (SCL) and phasic (SCR) components, which can be examined separately or jointly. Tonic SCL, a slowly fluctuating baseline, can be tracked across extended intervals (e.g., minutes), whereas SCRs are transient, event-linked increases in conductance observed shortly after stimuli or manipulations. Specifically, SCL can drift over time due to factors such as ambient temperature or general habituation. Both SCL and SCR are typically positively skewed and leptokurtic, and log transformations are often employed to normalize the data (see section 2.5.2 of Boucsein, 2012).

EDA datasets are frequently dichotomized into responders (participants who show SCRs above a minimum amplitude on at least X% of trials) and nonresponders (those who rarely or never exceed that threshold). Roughly 20% of healthy adults fall in the latter group when a 0.01–0.05μS criterion is used (Society for Psychophysiological Research Ad Hoc Committee on Electrodermal Measures, 2012). From a GT perspective, making this classification outside the G study effectively stratifies the objects of measurement, resulting in two strata of persons, with trials nested within persons and persons nested within strata. Variance that might otherwise be attributed to *Persons* or *Persons* × *Trials* is partitioned differently, and the dependability coefficients you obtain apply only to the retained stratum (responders).

An alternative approach is to keep the full sample in the G study and let “responsiveness” emerge as score variation. Then, if the research question involves classifying individuals at a particular cut score, cut-score-specific D-study coefficients for that decision can be calculated (Brennan and Kane, 1977; Kane and Brennan, 1980). Doing so quantifies how reliably the measure separates responders from nonresponders without discarding data or respecifying the object facet. Only when the study’s target population is intentionally limited to responders (e.g., biofeedback training studies that require observable SCRs) should a separate G study be carried out within that stratum. Taken together, GT offers two reasonable approaches for handling low-reactive participants: (a) include them and evaluate dependability for absolute decisions at the nonresponse cut score, or (b) treat responder status as a higher-level facet and conduct a stratified (nested) analysis. Either approach is preferable to excluding data a priori, which can artificially narrow score variance and limit generalizability.

A researcher can investigate the effect of altering the number of trials, adjusting thresholds, or combining data across occasions to enhance reliability for distinguishing between responders and nonresponders or for quantifying the magnitude of EDA-based reactivity. GT approaches facilitate the assessment of the reliability of cut points or thresholds for distinguishing responders from nonresponders or for distinguishing response trials from nonresponse trials (e.g., Brennan, 2001; Vispoel et al., 2018). The use of GT could yield subject-level criterion scores that would provide the capability to identify both nonresponse trials and participant nonresponders based on observed data. Like other psychophysiological modalities, multilevel models are particularly well suited to estimating variance components of EDA data, given the likelihood of unbalanced observations, zero-inflation (if nonresponse trials are zeroed out), and other departures from normality. By embracing GT’s multifaceted framework, researchers can capitalize on more precise variance estimates, better understand construct-relevant variance, and optimize task designs to capture reliable indices of EDA.

## 5. Difference scores

Difference scores are discussed here because many measures of interest in psychophysiological research are operationalized as contrasts (e.g., condition differences, lateralized indices). In these contexts, the inferences depend on the reliability of a difference score. GT provides a straightforward framework for characterizing the reliability of difference scores and for evaluating how design choices (e.g., number of trials per condition) influence the resulting difference-score reliability. This section therefore extends the univariate GT concepts introduced above by addressing a common scoring practice in psychophysiological research.

The psychometric reliability of subtraction-based difference scores has been a longstanding concern in the literature (e.g., Llabre et al., 1991). For instance, the reliability of a difference score depends on the reliabilities of the constituent measurements and the correlation between them. Holding the reliabilities and variances of the constituent scores constant, higher positive correlations between constituent scores generally reduce the reliability of the observed difference scores.

In studies of ERPs, difference scores are used to isolate neural activity of interest, and there are two general types. One type of difference score attempts to isolate neural activities across conditions of interest, such as error minus correct trials or gain minus loss trials. By subtracting correct trials (subtrahend) from error trials (minuend), this purportedly isolates error-related neural activity from overall activity associated with response processing (Clayson et al., 2021a). Another type of difference score attempts to isolate neural activity within a single condition of interest, when the two scores are measured concurrently, such as when using a peak-to-peak amplitude difference score or when estimating ipsilateral vs. contralateral activity (e.g., N2pc or lateralized readiness potential). The formulas for estimating the internal reliability (CE) using a one-facet design or for estimating internal reliability (CE) or test-retest reliability (CS) using a two-facet design are shown in Tables 6 and 7.

When difference scores are measured concurrently (e.g., ipsilateral vs. contralateral), the error covariance can be estimated (e.g., $2\sigma_{XY}(pi,e)$, see Table 6), but when difference scores are not measured concurrently (e.g., error trials minus correct trials), the error covariance cannot be estimated—the two events are not considered naturally co-occurring. Therefore, the error covariances for measures not measured concurrently should be set to zero (e.g., $2\sigma_{XY}(pi,e)=0$, see Table 6).

When using difference scores with non-ERP psychophysiological modalities, the same considerations about reliability apply. For instance, researchers could calculate EDA difference scores by subtracting a baseline skin conductance level from a post-stimulus level to isolate a phasic response to a specific event or calculate ECG difference scores to quantify changes in heart rate or heart rate variability across conditions (e.g., stress vs. relaxation). As with ERP difference scores, the reliability of these difference scores depends on (a) the reliability of each constituent measure, (b) the correlation between the constituent measures, and (c) whether the difference is derived from concurrently measured signals or from separate conditions or time points. If the difference scores represent concurrently measured signals, error covariances can be estimated. However, when the scores are derived from non-concurrent measurements (e.g., stress vs. relaxation), the error covariances are set to zero.

### 5.1. Explanations of low reliability of difference scores

Because the reliability of difference scores is a function of the individual reliabilities of its constituent scores and correlation between them (see Table 6 for formulas), low reliability for either or both scores and the overlap between them will reduce the possible ceiling for the reliability of the associated difference score. Another crucial point in evaluating the reliability of difference scores is understanding why there may be low between-person variance in those differences. If the true phenomenon itself exhibits little change (e.g., stable measures such as unchanging traits or behaviors), then the difference score’s variance can be extremely small, and standard reliability indices will, by definition, be low. The difference score reliability also would be undefined even when scores for individuals change but to the same degree across individuals. Notably, this does not imply that the underlying measurement is flawed per se. Rather, it means that when true change is negligible or the same across individuals, relying on the difference score’s reliability is effectively meaningless as a gauge of its validity (Rast and Hofer, 2014; Rogosa and Willett, 1983; Willett, 1989). In such instances, even a perfect measure can yield difference-score reliabilities that are close to zero or undefined simply because there is virtually no variance in the true signal for the difference score.

Conversely, correlated measurement error (such as carryover effects) can inflate the observed variance in difference scores—and with it, ironically, the reliability coefficient. Because such errors often remain consistent across occasions, they can masquerade as trait-like stability even though they reflect shared state variance. This inflation occurs because positively correlated errors across occasions inflate the split-half (or parallel forms) covariance far more than it augments the total-variance denominator (Edwards, 1994); systematic noise therefore drives conventional internal reliability indices upward (Rogosa and Willett, 1983). Consequently, when a difference score appears to show “acceptable” reliability, it is still essential to determine whether the coefficient reflects genuine inter-individual change or merely shared measurement error, or transient state variance, by directly examining the constituent measurements. In either scenario, conventional internal reliability indices might offer little insight into the cause of low (or high) reliability for difference scores: The crucial task is to determine the extent to which minimal variance arises from genuinely stable processes vs. measurement artifacts (or short-lived state processes). By examining complementary indices such as growth rate reliability (Willett, 1989) or by altering study designs to embed more intensive assessments (Rast and Hofer, 2014), researchers can better distinguish when difference scores have substantive value. Ultimately, theory and research aims, not omnibus statistical comparisons, should dictate whether a difference score is retained, refined, or replaced (Bedeian and Day, 1994).

Finally, when the goal is to evaluate difference-score reliability while retaining full information, such as variances and reliabilities, about each constituent score, multivariate GT designs provide a general solution by estimating variances and covariances of all constituent scores directly (see section 6). In this sense, subtraction-based difference scores can be viewed as one implementation of a broad framework that can be evaluated within multivariate GT.

## 6. GT multivariate and bifactor designs

When data consist of assessments that yield both composite and subcomponent scores, GT multivariate (Vispoel et al., 2024a, Vispoel et al., 2025; Vispoel et al., 2024b; Vispoel et al., 2024c; Vispoel et al., 2023a, 2023b, 2023c) and bifactor (Vispoel et al., 2025; Vispoel et al., 2023a, 2023b, 2023c; Vispoel et al., 2024b; Vispoel et al., 2022a, Vispoel et al., 2023a, 2023b, 2023c) designs can be analyzed to simultaneously partition observed score variance at both composite and subcomponent levels.^11^ In a multivariate design, each subcomponent could be treated as a fixed facet nested under the global (composite) score, allowing the researcher to estimate measurement error contributions for each subcomponent and then aggregate that information to derive reliability indices for the global score. Multivariate GT facilitates concurrent evaluation of subscale-level score generalizability and the derivation of correlation coefficients among those subscales corrected for multiple sources of error (e.g., item effects, person-by-occasion effects). For example, ERN and Pe amplitudes could be examined within a multivariate design when a researcher might wish to capture variance not just in a total error-monitoring index (a composite score of ERN and Pe amplitudes) but also in each of the two ERP scores, all while accounting for multiple sources of error such as trial-to-trial variability, session-to-session variability, or changes in participant state. Another example could be using EMG data from the NPU threat task that would facilitate estimating reliability for each experimental condition separately, while also providing covariances for the estimation of difference score reliability (i.e., difference score reliability estimation is handled within a multivariate design).

Bifactor GT designs further decompose universe-score variance into a general factor (shared across all subcomponents) and group factors specific to each subcomponent. This approach (a) clarifies the distinctiveness versus redundancy of subcomponent constructs, (b) produces indices showing how much variance in a subcomponent is unique, and (c) facilitates investigating whether certain subscales primarily reflect a general domain or also contain unique information. In ERN/Pe research, a bifactor GT design would identify measurement precision for an overall error-monitoring construct and distinguish variance that is common to both ERN and Pe (the general factor) from variance unique to each ERP (the group factors). In doing so, bifactor GT highlights the extent to which each subcomponent adds unique predictive value above and beyond the general factor.

## 7. Estimation of variance components

In GT, variance components are estimated to determine how different sources contribute to overall variability in the data. A common challenge within the GT framework is the way variance components are estimated. Researchers often estimate variance components using a random-effects ANOVA model where variance components are calculated using expected mean square (Brennan, 2001, 2010). Unfortunately, ANOVA is not well equipped to handle unbalanced data, a problem common to psychophysiological research (e.g., an unbalanced number of trials across participants due to artifact rejection procedures), that can result in negative variance components that could potentially render the computation of G and D coefficients meaningless. Alternatively, linear mixed models using restricted maximum likelihood estimation attempt to fix this problem by setting negative variance components to zero during the iteration process. However, this approach prevents the derivation of standard errors and confidence intervals (LoPilato et al., 2015). Thus, a researcher might not be able to infer the stability of the estimated variance components. Consequently, both approaches may yield inaccurate estimates of variance components that ultimately result in inaccurate reliability estimates.

As pointed out by LoPilato et al. (2015), Bayesian multilevel models offer a solution to these challenges. For one, Bayesian multilevel models do not result in negative variance component estimates when using proper prior constraints. Generally, the structure of Bayesian models does not allow realizations of parameter values outside of their defined space (for an example study comparing GT with congeneric univariate and bifactor model analyses, see Vispoel et al., 2022a). Particularly, variance components are strictly positive and a properly defined Bayesian model cannot yield negative estimates. This behavior is guided by the combination of the likelihood, which contains the functional model specification, and the choice of prior distributions for each parameter of interest, including variance components. The product of the likelihood and the priors yields posterior distributions that do not contain estimation artifacts, such as negative variances. Moreover, by defining prior distributions that reflect their beliefs about missing or excluded data points, researchers are able to provide reasonable starting points for the imputation of these values that yield more robust prediction models and consequently handle unbalanced data with greater flexibility (Klein et al., 2016). This is particularly useful when working with small datasets.

Traditional approaches to estimating variance components (e.g., ANOVA, linear mixed models) assume homoscedastic residuals for the conventional F-tests or likelihood-ratio tests used in inference; although heteroscedasticity-robust options are available, substantial departures from equal residual variances can still distort variance-component estimates, particularly in the small, unbalanced datasets common to psychophysiology (Clayson et al., 2019). The Bayesian multilevel modeling approach,^12^ in contrast, can explicitly model heteroscedasticity and estimate residual variances for each grouping variable (e.g., participants, conditions), placing those variances under a higher-level distribution. This multilevel “distribution of variances” improves point estimates, yields full uncertainty intervals, and facilitates treating heterogeneity of variances as a substantive feature of the data (one that may itself vary systematically across conditions or populations). In this way, the Bayesian framework accommodates unbalanced designs, preserves interpretability of variance components, and turns variance heterogeneity into an explanatory target instead of a statistical nuisance.

Unlike frequentist approaches, Bayesian models provide a flexible framework for defining the underlying family distribution of the data (e.g., non-Gaussian distributions). By accurately defining the appropriate distribution, researchers can make more reliable estimates of parameters, such as variance components, leading to more sound conclusions about the data. There are several software packages for estimating variance components and directly examining psychometric reliability using generalizability theory, but few of these general-purpose software packages^13^ can handle the unbalanced and multifaceted nature of psychophysiological data. To address these needs, the ERP Reliability Analysis Toolbox (ERA Toolbox; https://peclayson.github.io/ERA_Toolbox/) was designed with the needs of psychophysiologists in mind (Clayson et al., 2021a; Clayson et al., 2021c; Clayson et al., 2021d; Clayson and Miller, 2017a). The ERA Toolbox uses Bayesian multilevel models to estimate variance components of scores. However, the ERA toolbox only provides traditional estimates of group-level reliability of single events (Baldwin et al., 2015; Clayson and Miller, 2017a), internal reliability of difference scores (Clayson et al., 2021a), subject-level internal reliability (Clayson et al., 2021c), and group-level test-retest reliability of single events (Carbine et al., 2021; Clayson et al., 2021d). While the ERA Toolbox assumes a normal distribution of the underlying data, it could readily handle data from equal-length splits.

## 8. Example analysis of coefficients of equivalence

A ready example of the application of GT to ERP data uses the study design described in sections 3.1, 3.2, and 3.3, wherein ERN was recorded from participants while they completed flanker, Stroop, and go/no-go tasks (Holbrook et al., 2025; a replication of Meyer et al., 2013). A two-facet design (*Persons* × *Trials* × *Tasks*) was used to estimate CEs (i.e., internal reliability) of ERN scores to determine whether the internal reliability of ERN scores was similar across tasks. During the G study phase, *trials* was treated as a random facet, as ERN trials are considered exchangeable with one another, and *tasks* were treated as fixed to focus inferences on the three specific tasks. Variance components were estimated with a single multilevel location-scale model that preserved the crossed *Persons* × *Trials* structure within each task while treating task as a fixed facet. Although the outcome (ERN amplitude) is modeled univariately, explicitly including task in the model allows separate estimation of persons, trial, and residual variance components for each paradigm. This univariate-outcome, multi-facet specification thereby retains the ability to compare reliability coefficients across the three tasks without aggregating scores. Researchers could achieve equivalent results by running three independent *Persons* × *Trials* GT analyses (one per task), but the unified model offers a more parsimonious framework and facilitates direct statistical contrasts among tasks using the posterior parameter estimates.

As a first step, intraclass correlation coefficients (ICCs) were examined to provide a trial-independent estimate of reliability (Holbrook et al., 2025). The ICC characterizes how much of the total variance in ERN amplitude is from stable, between-person differences rather than item-level variance or error variance, and ICCs do not take into consideration the number of trials retained for averaging. Notably, ICCs were similar across the three tasks, suggesting that the ratio of universe-score (person-level) variance to total variance was largely consistent across tasks. However, ICCs cannot show whether one task yields more reliable scores once differences in the number of trials are taken into account.

Dependability coefficients diverged from the ICCs. Unlike the ICC, dependability coefficients incorporate the number of incorrect trials, which reduces the contribution of error variance to the dependability coefficient (e.g., see Table 2). Although each task included 420 trials, accuracy was highest during the go/no-go task (88%) and decreased in the order of Stroop (83%) and then flanker (79%). Considering that more mistakes were made during the flanker task than during the other two tasks, it would be expected that the flanker task yielded the highest dependability coefficients, which was indeed the case. More specifically, ERN scores from the flanker task yielded the highest dependability (ϕ=0.86;95% credible interval [CrI]: 0.82, 0.89) followed by dependability of the go/no-go task (ϕ=0.76;95% CrI: 0.70, 0.81) and then Stroop task (ϕ=0.77;95% CrI: 0.72, 0.82). Post-estimation contrasts confirmed that dependability was statistically higher during the flanker task than during the other two tasks, which yielded similar dependability estimates. Thus, tasks yielded similar ratios of person-level variance to total variance (ICCs) but exhibited different dependability estimates due to differences in the number of incorrect trials. In the Holbrook et al. (2025) study, the flanker task’s superior dependability stemmed from the greater number of usable incorrect trials than Stroop or go/no-go tasks rather than an intrinsically cleaner signal, making the flanker paradigm the most suitable of the three tested paradigms for assessing individual differences in ERN scores.

The juxtaposition of ICCs and dependability coefficients illustrates an important point: The three tasks similarly capture person-level signal per trial, but the flanker paradigm yields more usable incorrect trials and therefore a more dependable aggregate score. When a fixed number of trials was used across tasks, there were no statistical differences in dependability (Holbrook et al., 2025), but common practice is to use all available incorrect trials in analysis. Therefore, the flanker’s low accuracy makes it the best choice among the three tasks.

Rather than relying on a single indicator of consistency, a GT design for each task provides a systematic breakdown of how each source of error (task or trial) affects the dependability of ERP scores. This is especially valuable for multi-task ERP studies, as it allows researchers to (a) directly compare reliability across different paradigms, (b) pinpoint which tasks yield the most stable signals, and (c) ensure that measurement designs align with the specific goals of a study (e.g., identifying how many trials are needed for examinations of individual differences). In short, GT thus delivers a blueprint for designing ERP studies by highlighting where error arises and how design choices, such as task selection, affect reliability.

## 9. Conclusion

Psychophysiological measures offer unique insights into individual differences in cognition, affect, and behavior. However, the complexities inherent in these data, including varied distributions, unbalanced designs, and multiple sources of variance, can challenge traditional reliability frameworks. GT provides a flexible, multifaceted approach to understanding and optimizing psychometric reliability in these contexts. By parsing variance components across multiple facets (e.g., trials, occasions, tasks), researchers can readily identify sources of measurement error that compromise psychometric reliability, appropriately characterize psychometric reliability, and make informed decisions to improve study designs.

Illustrative applications of GT to EEG/ERP, ECG, EMG, and EDA data demonstrate that specifying facets and leveraging robust variance estimation methods can greatly enhance the utility of psychophysiological measures. In particular, Bayesian multilevel modeling offers a powerful avenue for variance component estimation, enabling researchers to incorporate prior information, handle unbalanced data, and account for heterogeneous variances across conditions. Beyond informing internal and retest reliability, GT approaches facilitate examinations of cut-score reliability and allow researchers to distinguish construct-relevant variance from task-specific or participant-specific sources.

An extension of GT for psychophysiological research could use multiverse analyses (Clayson, 2024a; Clayson et al., 2021b), wherein researchers systematically vary key data-processing and analytic decisions (e.g., artifact rejection thresholds, segmentation schemes, baseline corrections) to examine how robust GT-based reliability estimates are across plausible analytic pipelines. For example, after defining multiple reasonable ways of handling trials with artifacts or noise, the same GT design could be estimated for each data-processing “universe,” characterizing how changes in data-processing decisions impact variance components. This approach could reveal which decisions meaningfully affect reliability indices and which have negligible effects, thereby offering a clear picture of how measurement precision might shift across analytic variations.

Although it is important to use measurements with high reliability, reliability alone does not guarantee that a measure captures the construct it purports to assess. Construct validity often entails demonstrating a plausible causal or theoretical linkage between the attribute of interest and the measured signal (Borsboom, 2005; Borsboom and Mellenbergh, 2007; Borsboom et al., 2004). A given psychophysiological index (e.g., ERP component scores) may yield excellent reliability yet still fail to reflect a cognitive process that researchers assume it measures. Theoretical models that explain how changes in the supposed construct produce changes in the recorded biological activity need to be combined with rigorous reliability analysis (see Clayson, 2024b, 2025). Compelling evidence, such as convergent and discriminant validity with external measures (Cronbach and Meehl, 1955) and a clear conceptual framework (Borsboom et al., 2004), is essential before concluding that reliable psychophysiological measurements are valid for the intended purpose of measurement.

Ultimately, the comprehensive and nuanced perspective afforded by GT can increase reproducibility and interpretability of psychophysiological research. By improving the quality and consistency of psychophysiological measurements, researchers can more confidently link biological signals to psychological phenomena, thereby strengthening the foundation for understanding individual differences in healthy and clinical populations.

## Figures and Tables

**Table 1:** Definitions of terms (listed alphabetically) used in generalizability theory (GT).

GT Term	Conceptual Definition
Condition	A systematic way in which a measurement situation can vary (e.g., different events of interest), analogous to a “level” of a factor in an ANOVA. Each condition can contribute uniquely to the observed variance.
Coefficient of Equivalence (CE)	A reliability coefficient indicating how consistently a measure performs across different but parallel items/trials, forms, or versions administered at approximately the same time. In classical test theory, equivalence primarily focuses on whether the various forms yield similar scores under similar conditions. In GT, equivalence is defined more flexibly, sometimes referring to random equivalence, depending on how forms or items are conceptualized within the measurement design.
Coefficient of Trial Equivalence and Stability (CES)	A reliability coefficient that simultaneously accounts for two major sources of measurement error: equivalence (consistency across parallel items/trials, forms, or versions) and stability (consistency over time). The CES estimates the proportion of variance in observed scores attributable to the *true* person variance, relative to the combined variance from all relevant error sources.
Coefficient of Stability (CS)	A reliability coefficient indicating how consistently the same measure performs when administered over multiple occasions. “Stability” focuses on test–retest reliability (i.e., whether scores remain similar across time).
Decision (D) Study	A phase in GT used to design and optimize the actual measurement procedure for a specific purpose (e.g., how many items, how many sessions). The D study uses variance components (estimated in the G study) to project reliability under different measurement designs.
Dependability (ϕ)	A reliability coefficient indicating how well the measurement reflects each individual’s absolute level on the construct across the conditions of the universe of generalization. ϕ incorporates all measured error sources (including main facet effects).
Facet	An aspect or characteristic of the measurement situation that can vary (e.g., items, observers, or sessions), analogous to a factor in an ANOVA. Each facet contributes to score variation that can be estimated and controlled when it is observed at multiple levels; when only one level is observed, its variance component is not identifiable and the facet is treated as hidden (unmodeled). Facets may be treated as fixed or random depending on the intended universe of generalization.
Fixed Facet	A facet for which all possible conditions of interest are used (i.e., you have no intention to generalize beyond those specific conditions). For example, if the same set of conditions is always used (congruent vs. incongruent trial types), the item facet is considered “fixed.”
Generalizability $\left(\text{E}\rho^{2}\right)$	A reliability coefficient indicating how well the measurement preserves relative differences among individuals across the conditions of the universe of generalization. $\text{E}\rho^{2}$ excludes main facet effects from the error term.
Generalizability Study	A phase in GT used to estimate how different facets (and their interactions) contribute to total score variance. The G study partitions sources of error to identify where and how measurement inconsistencies arise.
Hidden Facet	A potential source of measurement variance often with only one level that cannot be modeled (e.g., hardware, occasion in a single-session study).
Object of Measurement	The entity (usually persons) being measured or observed. In psychophysiology, participants are typically the object of measurement, sampled from a larger population.
Random Facet	A facet for which the conditions are considered representative samples from a larger population of possible conditions (e.g., sample of error trials from all possible error trials). The aim is to generalize findings beyond the specific conditions measured.
Universe of Admissible Observations	The complete set of all potential measurement conditions and how they are defined or combined in a study. It represents every facet level that could be used or varied in collecting data.
Universe of Generalization	The set of conditions (from any facet) to which the researcher *intends* to generalize. These conditions define the boundary of interest (e.g., all items of a certain type, all measurement occasions across a month).
Universe Score	A person’s average (or expected) score across all conditions in the universe of generalization, analogous to the “true score” in classical test theory.

Note: ANOVA = analysis of variance.

**Table 2:** Generalizability theory models, partitioning, and score consistency indices for a one-facet design (Persons × Trials).

Design and Characteristic		Formula		
		$X_{pi}=$	Score of a person on a given trial =	
		μ	Mean across persons and trials	
		$+\left(\mu_{p}-\mu \right)$	+ persons effect (p)	
		$+\left(\mu_{i}-\mu \right)$	+ trials effect (i)	
		$+\left(X_{pi}-\mu_{p}-\mu_{i}+\mu \right)$	+ persons × trials interaction (p × i) and other error	
Partitioning of Variance	Individual Score	$\sigma_{X}^{2}=\sigma_{p}^{2}+\sigma_{i}^{2}+\sigma_{pi,e}^{2}$	Mean Score	$\sigma_{X_{pI}}^{2}=\sigma_{p}^{2}+\frac{\sigma_{pi,e}^{2}}{n_{i}^{\prime }}$
Error Variances	Relative	$\frac{\sigma_{pi,e}^{2}}{n_{i}^{\prime }}$	Absolute	$\frac{\sigma_{pi,e}^{2}}{n_{i}^{\prime }}+\frac{\sigma_{i}^{2}}{n_{i}^{\prime }}$
Standard Error of Measurement	Relative	$\sqrt{\frac{\sigma_{pi,e}^{2}}{n_{i}^{\prime }}}$	Absolute	$\sqrt{\frac{\sigma_{pi,e}^{2}}{n_{i}^{\prime }}+\frac{\sigma_{i}^{2}}{n_{i}^{\prime }}}$
Global coefficients	G-coefficient	$\frac{\sigma_{p}^{2}}{\sigma_{p}^{2}+\frac{\sigma_{pi,e}^{2}}{n_{i}^{\prime }}}$	D-Coefficient	$\frac{\sigma_{p}^{2}}{\sigma_{p}^{2}+\frac{\sigma_{pi,e}^{2}}{n_{i}^{\prime }}+\frac{\sigma_{i}^{2}}{n_{i}^{\prime }}}$
ICC	Relative	$\frac{\sigma_{p}^{2}}{\sigma_{p}^{2}+\sigma_{pi,e}^{2}}$	Absolute	$\frac{\sigma_{p}^{2}}{\sigma_{p}^{2}+\sigma_{pi,e}^{2}+\sigma_{i}^{2}}$
Cut-Score				$\frac{\sigma_{p}^{2}+{\left(\mu_{X}-C\right)}^{2}}{\sigma_{p}^{2}+{\left(\mu_{X}-C\right)}^{2}+\frac{\sigma_{pi,e}^{2}}{n_{i}^{\prime }}+\frac{\sigma_{i}^{2}}{n_{i}^{\prime }}}$

*Note*: Mean (μ) and variance (σ) expressed for a given person (p) and trial (i). The primes used with the ns represent the numbers of replicates (e.g., trials) within different contexts. In practice, estimates would be substituted for the parameters shown with appropriate corrections made for possible bias. ICC = intraclass correlation coefficient.

**Table 3:** Generalizability theory models, partitioning, and score consistency indices for a two-facet design (Persons × Trials × Occasions).

Design and Characteristic		Formula		
		$X_{\text{pio}}=$	Score of a person on a given trial and occasion =	
		μ	Mean across persons, trials, and occasions	
		$+\left(\mu_{p}-\mu \right)$	+ persons effect (p)	
		$+\left(\mu_{i}-\mu \right)$	+ trials effect (i)	
		$+\left(\mu_{o}-\mu \right)$	+ occasions effect (o)	
		$+\mu_{pi}-\mu_{p}+\mu_{i}+\mu$	+ persons × items effect (p × i)	
		$+\mu_{po}-\mu_{p}+\mu_{o}+\mu$	+ persons × occasions effect (p × o)	
		$+\mu_{oi}-\mu_{o}+\mu_{i}+\mu$	+ occasions × trials effect (o × i)	
		$+X_{pio}-\mu_{po}-\mu_{pi}-\mu_{oi}+\mu_{p}+\mu_{o}+\mu_{i}-\mu$	+ persons × trials × occasions interaction (p × i × o) and other error	
Partitioning of Variance	Individual Score	$\sigma_{X_{pio}}^{2}=\sigma_{p}^{2}+\sigma_{po}^{2}+\sigma_{pi}^{2}+\sigma_{poi,e}^{2}+\sigma_{o}^{2}+\sigma_{i}^{2}+\sigma_{oi}^{2}$	Mean Score	$\sigma_{X_{pIO}}^{2}=\sigma_{p}^{2}+\frac{\sigma_{po}^{2}}{n_{o}^{\prime }}+\frac{\sigma_{pi}^{2}}{n_{i}^{\prime }}+\frac{\sigma_{poi,e}^{2}}{n_{o}^{\prime }n_{i}^{\prime }}$
Error Variances	Relative	$\frac{\sigma_{pi}^{2}}{n_{i}^{\prime }}+\frac{\sigma_{po}^{2}}{n_{o}^{\prime }}+\frac{\sigma_{poi,e}^{2}}{n_{o}^{\prime }n_{i}^{\prime }}$	Absolute	$\frac{\sigma_{pi}^{2}}{n_{i}^{\prime }}+\frac{\sigma_{po}^{2}}{n_{o}^{\prime }}+\frac{\sigma_{poi,e}^{2}}{n_{o}^{\prime }n_{i}^{\prime }}+\frac{\sigma_{o}^{2}}{n_{o}^{\prime }}+\frac{\sigma_{i}^{2}}{n_{i}^{\prime }}+\frac{\sigma_{oi}^{2}}{n_{o}^{\prime }n_{i}^{\prime }}$
Standard Error of Measurement	Relative	$\sqrt{\frac{\sigma_{po}^{2}}{n_{o}^{\prime }}+\frac{\sigma_{pi}^{2}}{n_{i}^{\prime }}+\frac{\sigma_{poi,e}^{2}}{n_{o}^{\prime }n_{i}^{\prime }}}$	Absolute	$\sqrt{\frac{\sigma_{po}^{2}}{n_{o}^{\prime }}+\frac{\sigma_{pi}^{2}}{n_{i}^{\prime }}+\frac{\sigma_{poi,e}^{2}}{n_{o}^{\prime }n_{i}^{\prime }}+\frac{\sigma_{o}^{2}}{n_{o}^{\prime }}+\frac{\sigma_{i}^{2}}{n_{i}^{\prime }}+\frac{\sigma_{oi}^{2}}{n_{o}^{\prime }n_{i}^{\prime }}}$
CE	G-coefficient	$\frac{\sigma_{p}^{2}+\frac{\sigma_{po}^{2}}{n_{o}^{\prime }}}{\sigma_{p}^{2}+\frac{\sigma_{po}^{2}}{n_{o}^{\prime }}+\frac{\sigma_{pi}^{2}}{n_{i}^{\prime }}+\frac{\sigma_{poi,e}^{2}}{n_{o}^{\prime }n_{i}^{\prime }}}$	D-Coefficient	$\frac{\sigma_{p}^{2}+\frac{\sigma_{po}^{2}}{n_{o}^{\prime }}}{\sigma_{p}^{2}+\frac{\sigma_{po}^{2}}{n_{o}^{\prime }}+\frac{\sigma_{pi}^{2}}{n_{i}^{\prime }}+\frac{\sigma_{poi,e}^{2}}{n_{o}^{\prime }n_{i}^{\prime }}+\frac{\sigma_{i}^{2}}{n_{i}^{\prime }}+\frac{\sigma_{oi}^{2}}{n_{o}^{\prime }n_{i}^{\prime }}}$
ICC	Relative	$\frac{\sigma_{p}^{2}+\sigma_{po}^{2}}{\sigma_{p}^{2}+\sigma_{po}^{2}+\sigma_{pi}^{2}+\sigma_{poi,e}^{2}}$	Absolute	$\frac{\sigma_{p}^{2}+\sigma_{po}^{2}}{\sigma_{p}^{2}+\sigma_{po}^{2}+\sigma_{pi}^{2}+\sigma_{poi,e}^{2}+\sigma_{i}^{2}+\sigma_{oi}^{2}}$
CS	G-coefficient	$\frac{\sigma_{p}^{2}+\frac{\sigma_{pi}^{2}}{n_{i}^{\prime }}}{\sigma_{p}^{2}+\frac{\sigma_{po}^{2}}{n_{o}^{\prime }}+\frac{\sigma_{pi}^{2}}{n_{i}^{\prime }}+\frac{\sigma_{poi,e}^{2}}{n_{o}^{\prime }n_{i}^{\prime }}}$	D-Coefficient	$\frac{\sigma_{p}^{2}+\frac{\sigma_{pi}^{2}}{n_{i}^{\prime }}}{\sigma_{p}^{2}+\frac{\sigma_{po}^{2}}{n_{o}^{\prime }}+\frac{\sigma_{pi}^{2}}{n_{i}^{\prime }}+\frac{\sigma_{poi,e}^{2}}{n_{o}^{\prime }n_{i}^{\prime }}+\frac{\sigma_{o}^{2}}{n_{o}^{\prime }}+\frac{\sigma_{oi}^{2}}{n_{o}^{\prime }n_{i}^{\prime }}}$
ICC	Relative	$\frac{\sigma_{p}^{2}+\sigma_{pi}^{2}}{\sigma_{p}^{2}+\sigma_{po}^{2}+\sigma_{pi}^{2}+\sigma_{pio,e}^{2}}$	Absolute	$\frac{\sigma_{p}^{2}+\sigma_{pi}^{2}}{\sigma_{p}^{2}+\sigma_{po}^{2}+\sigma_{pi}^{2}+\sigma_{poi,e}^{2}+\sigma_{o}^{2}+\sigma_{oi}^{2}}$
CES	G-coefficient	$\frac{\sigma_{p}^{2}}{\sigma_{p}^{2}+\left(\frac{\sigma_{po}^{2}}{n_{o}^{\prime }}+\frac{\sigma_{pi}^{2}}{n_{i}^{\prime }}+\frac{\sigma_{poi,e}^{2}}{n_{o}^{\prime }n_{i}^{\prime }}\right)}$	D-Coefficient	$\frac{\sigma_{p}^{2}}{\sigma_{p}^{2}+\left(\frac{\sigma_{po}^{2}}{n_{o}^{\prime }}+\frac{\sigma_{pi}^{2}}{n_{i}^{\prime }}+\frac{\sigma_{poi,e}^{2}}{n_{o}^{\prime }n_{i}^{\prime }}+\frac{\sigma_{o}^{2}}{n_{o}^{\prime }}+\frac{\sigma_{i}^{2}}{n_{i}^{\prime }}+\frac{\sigma_{oi}^{2}}{n_{o}^{\prime }n_{i}^{\prime }}\right)}$
ICC	Relative	$\frac{\sigma_{p}^{2}}{\sigma_{p}^{2}+\left(\sigma_{po}^{2}+\sigma_{pi}^{2}+\sigma_{poi,e}^{2}\right)}$	Absolute	$\frac{\sigma_{p}^{2}}{\sigma_{p}^{2}+\left(\sigma_{po}^{2}+\sigma_{pi}^{2}+\sigma_{poi,e}^{2}+\sigma_{o}^{2}+\sigma_{i}^{2}+\sigma_{oi}^{2}\right)}$
Cut-Score				$\frac{\sigma_{p}^{2}+{\left(\mu_{X}-C\right)}^{2}}{\sigma_{p}^{2}+{\left(\mu_{X}-C\right)}^{2}+\left(\frac{\sigma_{po}^{2}}{n_{o}^{\prime }}+\frac{\sigma_{pi}^{2}}{n_{i}^{\prime }}+\frac{\sigma_{poi,e}^{2}}{n_{o}^{\prime }n_{i}^{\prime }}+\frac{\sigma_{o}^{2}}{n_{o}^{\prime }}+\frac{\sigma_{i}^{2}}{n_{i}^{\prime }}+\frac{\sigma_{oi}^{2}}{n_{o}^{\prime }n_{i}^{\prime }}\right)}$

*Note*: Mean (μ) and variance (σ) expressed for a given person (p), trial (i), and occasion (o). The primes used with the ns represent the numbers of replicates (e.g., trials) within different contexts. In practice, estimates would be substituted for the parameters shown with appropriate corrections made for possible bias. CE = coefficient of equivalence; CS = coefficient of stability; CES = coefficient of trial equivalence and stability; ICC = intraclass correlation coefficient.

**Table 4:** Generalizability theory models, partitioning, and score consistency indices for a one-facet design using data splits (Persons × (Trials:Splits)).

Design and Characteristic		Formula		
Partitioning of Variance	Individual Score	$\sigma_{X_{p(i:s)}}^{2}=\sigma_{p}^{2}+\sigma_{ps}^{2}+\sigma_{p(i:s),e}^{2}+\sigma_{s}^{2}+\sigma_{\left(i:s\right)}^{2}$	Mean Score	$\sigma_{X_{p(I:S)}}^{2}=\sigma_{p}^{2}+\frac{\sigma_{ps}^{2}}{n_{s}^{\prime }}+\frac{\sigma_{p(i:s),e}^{2}}{n_{i\;per\;\text{split}}^{\prime }\;n_{s}^{\prime }}$
Error Variances	Relative	$\frac{\sigma_{ps,e}^{2}}{n_{s}^{\prime }}+\frac{\sigma_{p(i:s),e}^{2}}{n_{i\;per\;\text{split}}^{\prime }\;n_{s}^{\prime }}$	Absolute	$\frac{\sigma_{ps}^{2}}{n_{s}^{\prime }}+\frac{\sigma_{p(i:s),e}^{2}}{n_{i\;per\;\text{split}\;}^{\prime }n_{s}^{\prime }}+\frac{\sigma_{s}^{2}}{n_{s}^{\prime }}+\frac{\sigma_{\left(i:s\right)}^{2}}{n_{i\;per\;\text{split}\;}^{\prime }n_{s}^{\prime }}$
Standard Error of Measurement	Relative	$\sqrt{\frac{\sigma_{ps,e}^{2}}{n_{s}^{\prime }}+\frac{\sigma_{p(i:s),e}^{2}}{n_{i\;per\;\text{split}}^{\prime }\;n_{s}^{\prime }}}$	Absolute	$\sqrt{\frac{\sigma_{ps}^{2}}{n_{s}^{\prime }}+\frac{\sigma_{p(i:s),e}^{2}}{n_{i\;per\;\text{split}\;}^{\prime }n_{s}^{\prime }}+\frac{\sigma_{s}^{2}}{n_{s}^{\prime }}+\frac{\sigma_{\left(i:s\right)}^{2}}{n_{i\;per\;\text{split}\;}^{\prime }n_{s}^{\prime }}}$
Global coefficients	G-coefficient	$\frac{\sigma_{p}^{2}}{\sigma_{p}^{2}+\frac{\sigma_{ps,e}^{2}}{n_{s}^{\prime }}+\frac{\sigma_{p(i:s),e}^{2}}{n_{i\;per\;\text{split}}^{\prime }\;n_{s}^{\prime }}}$	D-Coefficient	$\frac{\sigma_{p}^{2}}{\sigma_{p}^{2}+\left(\frac{\sigma_{ps}^{2}}{n_{s}^{\prime }}+\frac{\sigma_{p(i:s),e}^{2}}{n_{i\;per\;\text{split}\;}^{\prime }n_{s}^{\prime }}+\frac{\sigma_{s}^{2}}{n_{s}^{\prime }}+\frac{\sigma_{\left(i:s\right)}^{2}}{n_{i\;per\;\text{split}\;}^{\prime }n_{s}^{\prime }}\right)}$
ICC	Relative	$\frac{\sigma_{p}^{2}}{\sigma_{p}^{2}+\sigma_{ps,e}^{2}+\sigma_{p(i:s),e}^{2}}$	Absolute	$\frac{\sigma_{p}^{2}}{\sigma_{p}^{2}+\left(\sigma_{ps}^{2}+\sigma_{p(i:s),e}^{2}+\sigma_{s}^{2}+\sigma_{p(i:s)}^{2}\right)}$
Cut-Score				$\frac{\sigma_{p}^{2}+{\left(\mu_{X}-C\right)}^{2}}{\sigma_{p}^{2}+{\left(\mu_{X}-C\right)}^{2}+\left(\frac{\sigma_{ps}^{2}}{n_{s}^{\prime }}+\frac{\sigma_{p(i:s),e}^{2}}{n_{i\;per\;\text{split}\;}^{\prime }n_{s}^{\prime }}+\frac{\sigma_{s}^{2}}{n_{s}^{\prime }}+\frac{\sigma_{\left(i:s\right)}^{2}}{n_{i\;per\;\text{split}\;}^{\prime }n_{s}^{\prime }}\right)}$

*Note*: Mean (μ) and variance (σ) expressed for a given person (p) and trial (i). The primes used with the ns represent the numbers of replicates (e.g., trials) within different contexts. In practice, estimates would be substituted for the parameters shown with appropriate corrections made for possible bias. ICC = intraclass correlation coefficient.

**Table 5:** Generalizability theory models, partitioning, and score consistency indices for a two-facet design (Persons × (Trials:Splits) × Occasions).

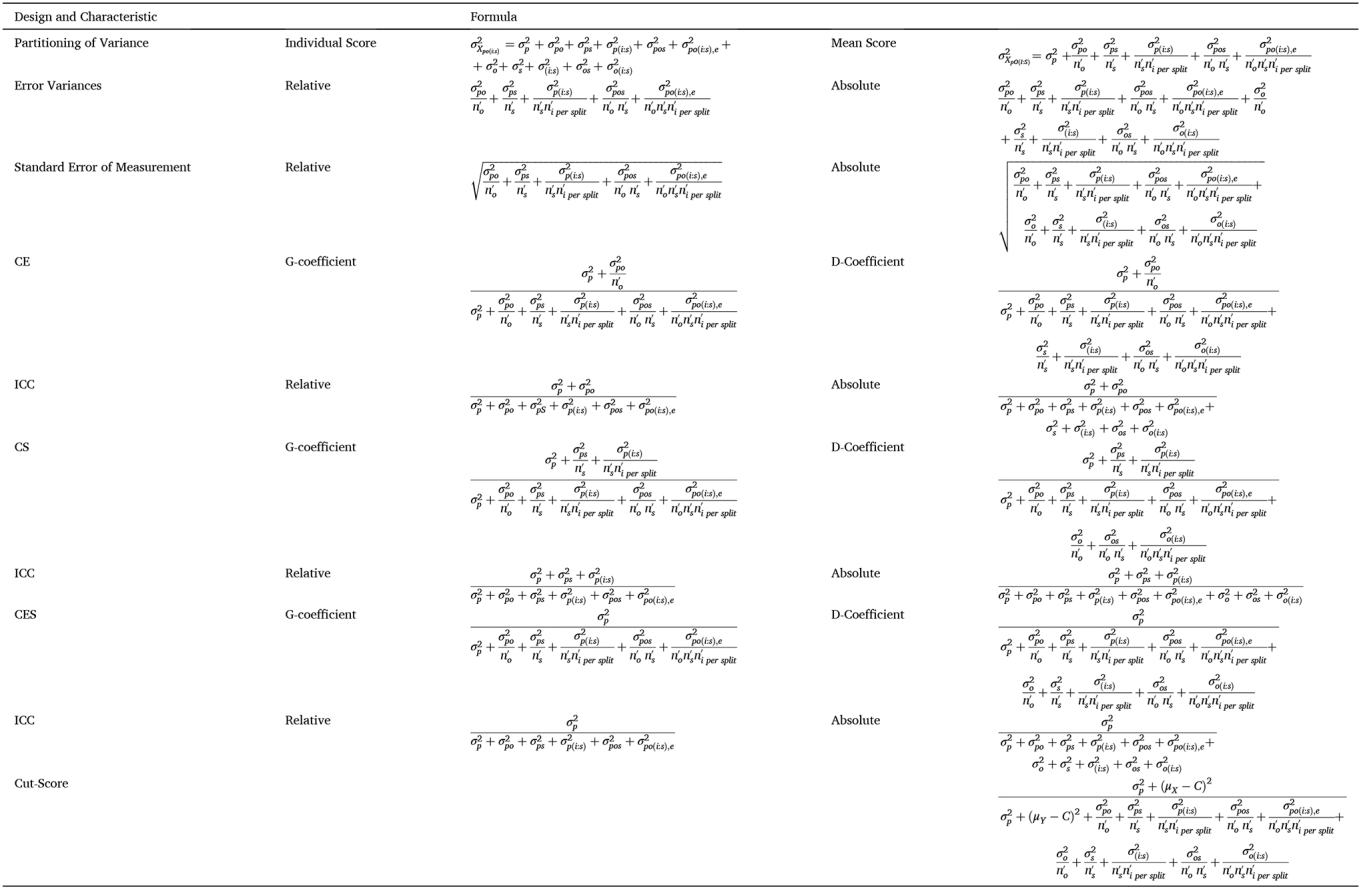

*Note*: Mean (μ) and variance (σ) expressed for a given person (p), trial (i), and occasion (o). The primes used with the ns represent the numbers of replicates (e.g., trials) within different contexts. In practice, estimates would be substituted for the parameters shown with appropriate corrections made for possible bias. CE = coefficient of equivalence; CS = coefficient of stability; CES = coefficient of trial equivalence and stability; ICC = intraclass correlation coefficient.

**Table 6:** Generalizability theory formulas for difference scores.

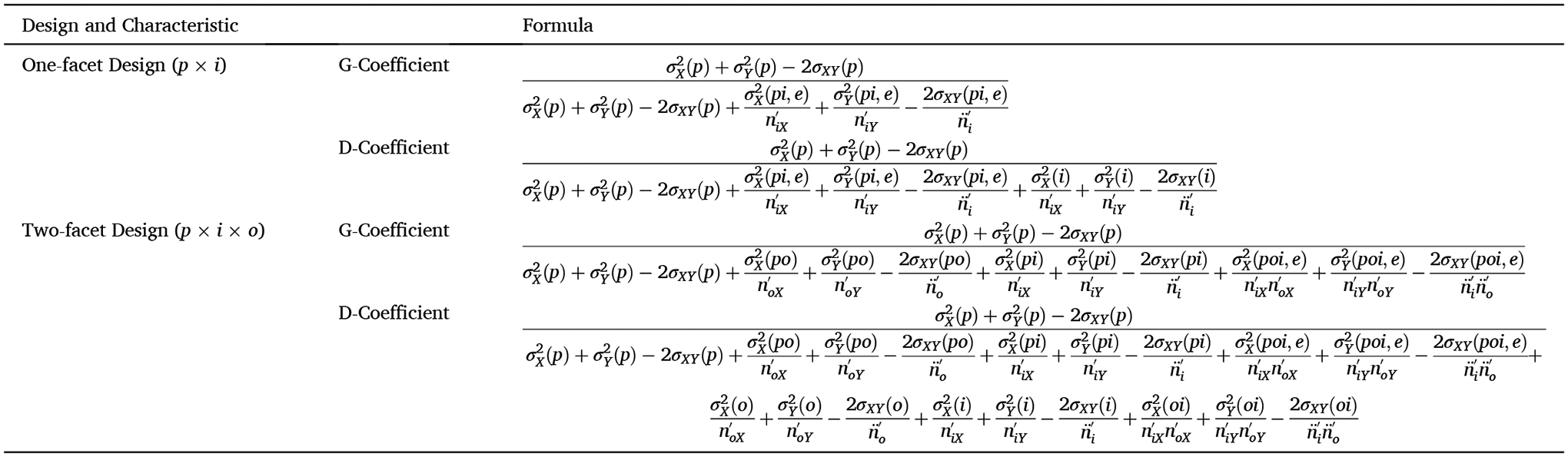

Note: Variance $\left(\sigma^{2}\right)$ expressed for a given person (p) and trial (i). X and Y refer to two separate measures (e.g., correct trials and error trials). Formulas for the two-facet design represent coefficients of equivalence and stability. To estimate coefficients of equivalence for the two-facet design, the following should be added to the numerator: $\frac{\sigma_{X}^{2}(po)}{n_{oX}^{\prime }}+\frac{\sigma_{Y}^{2}(po)}{n_{oY}^{\prime }}-\frac{2\sigma_{XY}(po)}{{\ddot{n}}_{o}^{\prime }}$. To estimate coefficients of stability for the two-facet design, the following should be added to the numerator: $\frac{\sigma_{X}^{2}(pi)}{n_{iX}^{\prime }}+\frac{\sigma_{Y}^{2}(pi)}{n_{iY}^{\prime }}-\frac{2\sigma_{XY}(pi)}{{\ddot{n}}_{i}^{\prime }}$. In practice, estimates would be substituted for the parameters shown with appropriate corrections made for possible bias. For covariance terms, the harmonic mean $\left(\ddot{n}\right)$ of the number of included observations for X and Y.

**Table 7:** Generalizability theory formulas for difference scores using data splits.

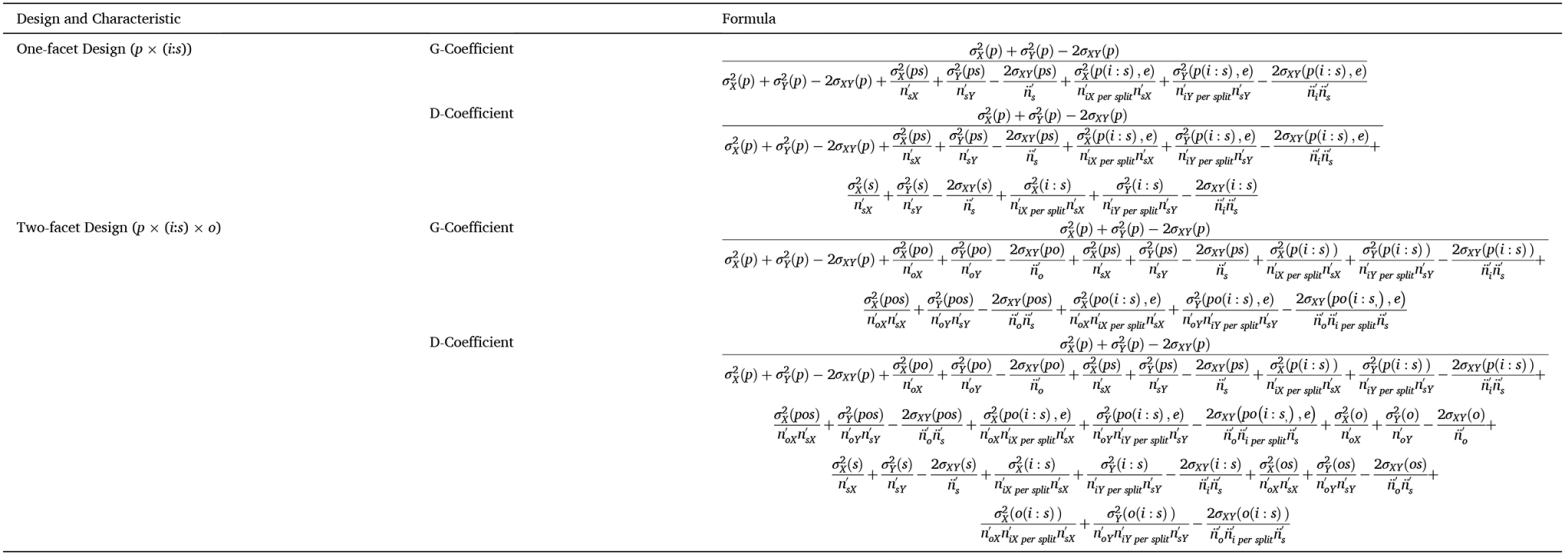

*Note*: Variance $\left(\sigma^{2}\right)$ expressed for a given person (p) and trial (i). X and Y refer to two separate measures (e.g., correct trials and error trials). Formulas for the two-facet design represent coefficients of equivalence and stability. To estimate coefficients of equivalence for the two-facet design, the following should be added to the numerator: $\frac{\sigma_{X}^{2}(po)}{n_{oX}^{\prime }}+\frac{\sigma_{Y}^{2}(po)}{n_{oY}^{\prime }}-\frac{2\sigma_{XY}\left(po\right)}{{\ddot{n}}_{o}^{\prime }}$. To estimate coefficients of stability for the two-facet design, the following should be added to the numerator: $\frac{\sigma_{X}^{2}(i:s)}{n_{iX\;\text{per}\;\text{split}}^{\prime }n_{sX}^{\prime }}+\frac{\sigma_{Y}^{2}(i:s)}{n_{iY\;\text{per}\;\text{split}\;}^{\prime }n_{sY}^{\prime }}-\frac{2\sigma_{XY}(p\left(i:s)\right)}{{\ddot{n}}_{i}^{\prime }{\ddot{n}}_{s}^{\prime }}$. In practice, estimates would be substituted for the parameters shown with appropriate corrections made for possible bias. For covariance terms, the harmonic mean $\left(\ddot{n}\right)$ is used for the number of observations included in splits for conditions X and Y.

## Data Availability

No data was used for the research described in the article.

## Footnotes

1 A comparison of GT and classical test theory for psychophysiological researchers is provided elsewhere (Baldwin et al., 2015; Clayson, 2024b; Clayson and Miller, 2017a, 2017b). A broad treatment is provided in Vispoel et al. (2018).

2 Trials are treated as synonymous with items. The same formulas and relationships would apply to either. This is done to align with other treatments of GT (e.g., Brennan, 2001; Vispoel et al., 2018).

3 A multivariate design could be informative for this type of study design by allowing each trial type and any potential composite scores (e.g., difference scores) to be modeled separately (see section 6 for more on multivariate GT). For example, ERN scores from Stroop, flanker, and go/no-go tasks could be analyzed as distinct subscales, or combined into composites if metrics are comparable (Le et al., 2009). Even if a composite is not of interest, ERN scores can still be treated separately within a multivariate framework or nested univariate designs.

4 Averaging all trials together is meant to reflect how ERP scores are typically used. Although ERN and Pe scores can change over the course of a single task or across an entire battery of tasks (Clayson et al., 2025a), the modal approach to analyzing ERP components is to average all trials together and treat the underlying construct as unidimensional. Therefore, the modal approach to analyzing ERP component scores is to treat each trial score as entirely exchangeable with any other trial score.

5 When the substantive claim is intended to generalize beyond a specific task (e.g., when interpreting ERN/Pe as indicators of error monitoring that should be observed across paradigms), *task* can be conceptualized as a random facet, such that the universe of generalization explicitly includes plausible levels of *task* (e.g., flanker, go/no-go, Stroop). In a generalizability framework, modeling *task* requires multiple tasks to be administered to each person. Treating *task* as fixed is most appropriate when the claim about reliability is intentionally specific to the tasks used (e.g., evaluating a specific task protocol, directly comparing specific paradigms, defining the construct in task-specific terms). When multiple paradigms are intended to define a broader universe of generalization, tasks should ideally yield comparable scores on a common metric; when tasks yield non-comparable outcomes, multivariate generalizability designs are required (see section 6). Importantly, single-task studies implicitly render *task* a hidden facet, and therefore *task* cannot be modeled as an estimable facet in the design. Within a single paradigm, replication typically occurs over trials, which can be modeled as facets; this supports generalization over those task-specific sampling units, but not over non-administered tasks.

6 Brennan et al. (2022) extend the standard multivariate generalizability framework (see section 6 for more on multivariate GT) to handle designs in which each test section involves a unique set of random-effect structures, such as an ERP component amplitude that can be measured using different tasks or scalp channels. If each “level” of these measurement conditions is treated as though they share the same psychometric structure (e.g., ignoring the fact that certain tasks may require different approaches for scoring such as scoring N2, a scalp-recorded ERP component that shows different temporal characteristics across go/no-go and flanker tasks), there is a risk of underestimating variability of facets and, hence, inflating reliability estimates. By explicitly modeling separate sets of variance components for each facet, such as electrode-specific noise, researchers obtain more accurate conditional standard errors and dependability indices for the final ERP component score. This is particularly important in mixed-design psychophysiology experiments, where multiple tasks, conditions, or electrode clusters may all contribute unique forms of measurement error.

7 For detailed discussions of standardization and norming as they relate to ERP scores, see Clayson (2024a, 2024b, 2025). Although those discussions focus on ERP components, the principles are broadly applicable to other psychophysiological measures.

8 The typical approach is to average activity for the entire condition. Therefore, no special meaning is attributed to any particular point during a condition of the TSST.

9 Stratified objects of measurement in GT refer to cases where the object of measurement (e.g., participants) is nested within a higher-order grouping (e.g., diagnostic category), allowing variance to be estimated separately within each group rather than across the full sample. This approach is appropriate in clinical research, where participants are not randomly sampled across groups and are not exchangeable between them. The focus is typically on estimating reliability within each group, rather than generalizing reliability across groups.

10 Another approach that considers uneven splits in the estimation of reliability is shown in Table A1 of Appendix A in Vispoel et al. (2018). Essentially, first the reliability coefficient is estimated using the uneven splits, then the coefficient is weighted to align with the Raju split-half coefficient (Raju, 1977).

11 The cited papers and their supplementary material contain the relevant formulas and code for estimating reliability in GT multivariate and bifactor designs.

12 Rast and Clayson (in press) integrate GT with multilevel location-scale models to relax the homoscedastic-error assumption and model residual variance as systematically varying across persons and conditions. This yields condition-specific and person-specific reliability functions that can characterize reliability differences masked by pooled-error models. They also illustrate how D-study planning can be adapted to allocate additional trials to low-precision conditions or participants.

13 Two general-purpose software packages include mGENOVA (https://education.uiowa.edu/casma/computer-programs) and gtheory (Moore, 2016).
